# Chemistry, Chemotaxonomy and Biological Activity of the Latrunculid Sponges (Order Poecilosclerida, Family Latrunculiidae)

**DOI:** 10.3390/md19010027

**Published:** 2021-01-09

**Authors:** Fengjie Li, Michelle Kelly, Deniz Tasdemir

**Affiliations:** 1 GEOMAR Centre for Marine Biotechnology (GEOMAR-Biotech), Research Unit Marine Natural Products Chemistry, GEOMAR Helmholtz Centre for Ocean Research Kiel, Am Kiel-Kanal 44, 24106 Kiel, Germany; fli@geomar.de; 2 Coast and Oceans National Centre, National Institute of Water and Atmospheric Research (NIWA) Ltd., P.O. Box 109-695, Newmarket, Auckland 1149, New Zealand; michelle.kelly@niwa.co.nz; 3 Faculty of Mathematics and Natural Sciences, Kiel University, Christian-Albrechts-Platz 4, 24118 Kiel, Germany

**Keywords:** latrunculid sponge, Latrunculiidae, pyrroloiminoquinone alkaloid, callipeltin, bioactivity, taxonomy, chemotaxonomy, biosynthetic origin

## Abstract

Marine sponges are exceptionally prolific sources of natural products for the discovery and development of new drugs. Until now, sponges have contributed around 30% of all natural metabolites isolated from the marine environment. Family Latrunculiidae Topsent, 1922 (class Demospongiae Sollas, 1885, order Poecilosclerida Topsent, 1928) is a small sponge family comprising seven genera. Latrunculid sponges are recognized as the major reservoirs of diverse types of pyrroloiminoquinone-type alkaloids, with a myriad of biological activities, in particular, cytotoxicity, fuelling their exploration for anticancer drug discovery. Almost 100 pyrroloiminoquinone alkaloids and their structurally related compounds have been reported from the family Latrunculiidae. The systematics of latrunculid sponges has had a complex history, however it is now well understood. The pyrroloiminoquinone alkaloids have provided important chemotaxonomic characters for this sponge family. Latrunculid sponges have been reported to contain other types of metabolites, such as peptides (callipeltins), norditerpenes and norsesterpenes (trunculins) and macrolides (latrunculins), however, the sponges containing latrunculins and trunculins have been transferred to other sponge families. This review highlights a comprehensive literature survey spanning from the first chemical investigation of a New Zealand *Latrunculia* sp. in 1986 until August 2020, focusing on the chemical diversity and biological activities of secondary metabolites reported from the family Latrunculiidae. The biosynthetic (microbial) origin and the taxonomic significance of pyrroloiminoquinone related alkaloids are also discussed.

## 1. Introduction 

Marine organisms have an extraordinary track record as an invaluable source of novel secondary metabolites with unique chemical structures and a wide range of pharmacological activities that hold the key for the development of novel drugs [1,2]. Marine natural product (MNP) research began with investigation on the Bahamian sponge *Tectitethya crypta* (de Laubenfels, 1949) in the 1950s, yielding the nucleosides spongothymidine and spongouridine [3,4,5]. These metabolites served as the leading structures for the synthesis of the first marine-derived anticancer agent cytarabine (Ara-C^TM^) and antiviral agent vidarabine (Ara-A^TM^). Marine sponges are among the most primitive multicellular animals that have existed for 700–800 million years, with approximately 8900 described species distributed worldwide today [6]. Being sessile, sponges lack behavioral defenses and have evolved numerous structural and chemical defense strategies to defend themselves, e.g., the accumulation of toxic and/or antifeedant molecules [7]. Sponge structural components such as spicules, spongin, and chitin fibers all exhibit high defensive efficiency [8,9]. Sponges also produce a vast array of secondary metabolites featuring unprecedented carbon skeletons and multiple ecological functions, such as chemical defenses, which often underly their pharmacological activities [10,11,12,13]. It is well known that sponges possess a very rich microbiome that also contributes to the production of bioactive secondary metabolites [7,14,15]. Marine sponges have received the greatest attention of all marine organisms, and are recognized as the most prolific sources of MNPs, contributing to nearly 30% of all MNPs reported so far [16,17]. Currently, three out of 14 approved marine-derived drugs on the market have their origin in marine sponges, namely Halaven^®^ (eribulin mesylate), Vira-A^®^ (Ara-A), and Cytosar-U^®^ (Ara-C) [18]. Furthermore, the sponge-derived metabolite plocabulin, a new tubulin-binding agent originally obtained from the sponge *Lithoplocamia lithiostoides*, is currently undergoing phase II clinical trials for the treatment of solid tumors; eribulin, a synthetic analogue of halichondrin B, which originated from sponge *Halichondria okadai*, is undergoing phase I clinical trials for its new application as payloads of antibody–drug conjugates (ADC) [18]. 

**Figure 1:**
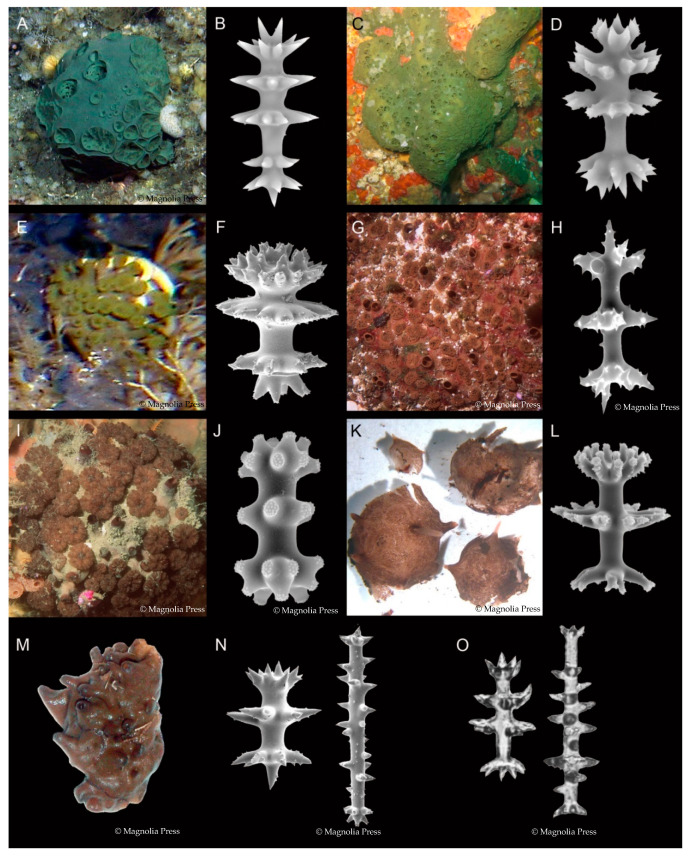
Living Latrunculiidae sponges and their diagnostic spicules. Genus *Strongylodesma* Levi, 1969, is not represented here because they lack the diagnostic discorhabds. Species in subgenus *Latrunculia* (*Latrunculia*) have anisodiscorhabd microscleres with six visibly distinct substructures: (**A**) *L.* (*L*.) *austini* Samaai, Gibbons & Kelly, 2006, Gulf of Alaska (reproduced from reference [21], Figure 3F, with permission from copyright holder); (**B**) Typical anisodiscorhabd, undescribed species from the Chatham Islands, New Zealand. Species in subgenus *Latrunculia* (*Biannulata*) have anisodiscorhabd microscleres with two distinct substructures around the shaft, the median and subsidiary whorls, between an undifferentiated manubrium and basal whorl, and the undifferentiated apical whorl and apex: (**C**) *L.* (*B.*) *kaakaariki* Alvarez, Bergquist & Battershill, 2002, Three Kings Islands, New Zealand (reproduced with permission from Crispin Middleton); (**D**) Typical anisodiscorhabd from the same species. Species in subgenus *Latrunculia* (*Uniannulata*) have anisodiscorhabd microscleres with only a single substructure around the shaft, the median whorl, between the manubrium and basal whorl, and the apical whorl and apex: (**E**) *L.* (*U.*) *oparinae* Samaai & Krasokhin, 2002, Aleutian Islands, Alaska (reproduced with permission from Robert Stone; reproduced from reference [21], Figure 8A, with permission from copyright holder); (**F**) Typical anisodiscorhabd from the same species. Species in genus *Cyclacanthia* are Latrunculiidae with acanthose isospinodiscorhabd microscleres which have a shaft bearing identical apical and basal substructures: (**G**) *Cyclacanthia bellae* Samaai, Gibbons, Kelly & Davies-Coleman, 2003 (reproduced with permission from the Coral Reef Research Foundation, Palau, Micronesia; reproduced from reference [26], Figure 3A, with permission from copyright holder); (**H**) Typical isospinodiscorhabd (reproduced from reference [21], Figure 1D, with permission from copyright holder). Species in genus *Tsitsikamma* are Latrunculiidae with acanthose isospinodiscorhabd microscleres which have a shaft bearing identical apical and basal substructures: (**I**) *Tsitsikamma favus* Samaai & Kelly, 2002, Algoa Bay, South Africa (reproduced with permission from the Coral Reef Research Foundation, Palau, Micronesia; reproduced from reference [41], Figure 8E, with permission from copyright holder); (**J**) Typical isochiadiscorhabd from the same species (reproduced from reference [21], Figure 1E, with permission from copyright holder). Species in genus *Bomba* are Latrunculiidae with unusual anisodiscorhabds that have only three substructures: (**K**) *Bomba endeavorensis* Kelly, Reiswig & Samaai, 2016, Endeavour Ridge, British Columbia (reproduced from reference [21], Figure 11A, with permission from copyright holder); (**L**) Typical anisodiscorhabd from the same species. Species in genus *Latrunclava* are Latrunculiidae with two microsclere forms, an anisodiscorhabd and several longer anisoconicorhabds that have structurally different apical and basal whorls, unlike *Sceptrella* species which also have two microsclere forms, but the longer amphiclad sceptre has identical apical and basal whorls: (**M**) *Latrunclava imago* Kelly, Reiswig & Samaai, 2016, Aleutian Islands (reproduced from reference [21], Figure 13A, with permission from copyright holder); (**N**) Anisodiscorhabd and long anisoconicorhabd (reproduced from reference [21], Figure 1G, with permission from copyright holder); (**O**) Anisodiscorhabd and amphiclad sceptre from *Sceptrella regalis* Schmidt, 1870 (reproduced from reference [21], Figure 1F, with permission from copyright holder). All images are reproduced with permission from the original photographers or from *Zootaxa*.

Latrunculiidae Topsent, 1922 [19] is a well-known and well-defined family of demosponges (class Demospongiae Sollas) within the order Poecilsoclerida Topsent, with an unusual amphipolar distribution; the 83 valid species presently recognized are largely distributed between the cold and temperate coastal shallow and deep-sea environments of: (1) South Africa; (2) New Zealand and Antarctica; (3) South America [20]; (4) the North Pacific Ocean, around the British Columbia, Alaskan, and Aleutian Islands, and the Kurile Islands, Sea of Okhotsk, Russia [21]. A few species are found between these primary regions, including the Southern Ocean, South Australia, New Caledonia, Japan, and the eastern Philippines. Latrunculid sponges have been recorded from the shallow subtidal zone down to 2500 m (*Bomba endeavourensis* Kelly, Reiswig & Samaai, 2016) [21]. Sponges of the family Latrunculiidae are all very similar in general morphology and skeletal architecture, having a differentiated aquiferous system of raised dome- to mushroom-shaped porefields and raised volcano-shaped oscules, and ranging in shape from spheres, to massive hemispheres, to pedunculate, to encrusting (Figure 1). The colour in life is characteristically rich chocolate to liver brown, sage to emerald green, deep turquoise to (rarely) purple and pink (Figure 1). The family Latrunculiidae now contains seven genera: *Latrunculia* du Bocage, 1869 (and three subgenera); *Sceptrella* Schmidt, 1870; *Strongylodesma* Levi, 1960; *Tsitsikamma* Samaai & Kelly, 2002; *Cyclacanthia* Samaai & Kelly, 2004; *Bomba* Kelly, Reiswig & Samaai, 2016; *Latrunclava* Kelly, Reiswig & Samaai, 2016 [21,22,23,24,25,26]. Species in the seven genera (and subgenera) are differentiated primarily on the morphology and ornamentation of the diagnostic microsclere—the anisodiscorhabd (Figure 1).

The chemistry of latrunculid sponges is dominated by (bis)pyrroloiminoquinone alkaloids (discorhabdins, tsitsikammamines) and structurally related derivatives (makaluvamines, batzellines, isobatzellines, etc.) that display various bioactivities; particularly anticancer, but also antimicrobial and antimalarial [27,28,29,30,31,32,33]. To date, almost 100 compounds belonging to this chemical family have been reported from latrunculid sponges, and their structure and activity relationships (SARs) and biosyntheses have been studied [30,34]. Taxonomic classification of this family has been complex. Chemical investigations on latrunculid sponges, especially the report of different (bis)pyrroloiminoquinone analogs, have provided significant chemotaxonomic evidence for phylogenetic revision of the family [21]. Pyrroloiminoquinone alkaloids are responsible for the brownish or greenish coloration of latrunculid sponges and play an important role in the chemical defense of the sponge [28,35]. Pyrroloiminoquinones are not exclusive to latrunculid sponges and have been reported from other resources, such as ascidians, different sponge families (Acarnidae, Stelligeridae, and Halichondriidae) and terrestrial microbes (myxomycetes) [36,37,38,39]. Hence, a microbial origin is being hypothesized for pyrroloiminoquinone-related alkaloids and the debate on their biosynthetic origin is on-going [40]. 

Latrunculid sponges also produce cyclodepsipeptides, the so-called callipeltins, that are characterized by the presence of numerous unusual amino acids and promising in vitro bioactivities (cytotoxicity, antifungal and anti-HIV) [42,43,44]. Some norditerpene or norsesterterpene cyclic peroxides (trunculins) and prominent cytotoxic macrolides (latrunculin A and other latrunculins) were also reported from sponge specimens that were loosely grouped in the sponge family Latrunculiidae [45,46,47]. Subsequent phylogenetic revisions assisted by marine natural product chemistry studies have reidentified the source sponges as *Dicarnus*, *Sigmaceptrella*, or *Negombata* species belonging to the sponge family Podospongiidae Laubenfels, 1936 [21,48,49,50,51]. 

Numerous studies have shown the chemical uniqueness and bioactivity potential of the latrunculid sponges, hence, a systematic review on this family appeals to a broad scientific community working in the fields of, e.g., marine natural product chemistry, drug discovery, medicinal chemistry, pharmacology, oncology, and sponge taxonomy. This review covers all chemical and biological investigations performed on the members of the family Latrunculiidae from 1986 to August 2020, covering 110 natural metabolites reported from five latrunculid sponge genera (*Latrunculia*, *Strongylodesma*, *Tsitsikamma*, *Sceptrella*, *Cyclacanthia*). The review also encompasses highly relevant subjects, such as the biosynthetic (microbial) origin of the metabolites and the (chemo)taxonomy of the latrunculid sponges.

## 2. Systematics of Latrunculiidae Sponges

- Class Demospongiae Sollas, 1885 [52];
- Subclass Heteroscleromorpha Cárdenas, Perez and Boury-Esnault, 2012 [53];
- Order Poecilosclerida Topsent, 1928 [54];
- Family Latrunculiidae Topsent, 1922 [19].

The name of the subclass follows the classification proposal by Morrow and Cárdenas (2015) [55]. The systematics of Latrunculiidae follows Samaai and Kelly (2002) [25], Samaai et al. (2003, 2004, 2006, 2009, 2012) [26,50,56,57,58], and Kelly et al. (2016) [21]. Samaai et al. (2004) and Kelly et al. (2016) illustrate the diagnostic microscleres that define the Latrunculiidae genera [21,26]. The genus *Strongylodesma* lacks the diagnostic discorhabd microsclere; the first temperate Australian species described was from New South Wales [59]. The discovery of the pyrroloquinoline alkaloid damirone A in the holotype of *S. australiense* strongly supported the assignment of this new species to *Strongylodesma*, rather than to *Batzella* Topsent, 1893 [49,60].

## 3. Chemical Investigations of Marine Sponges from Family Latrunculiidae

### 3.1. Genus Latrunculia

*Latrunculia* du Bocage, 1869 represents the largest genus of the family Latrunculiidae. It comprises over 30 valid species that can be further divided into three subgenera; *Biannulata*, *Latrunculia*, and *Uniannulata* [21]*. Latrunculia* is the best studied subgenus in terms of chemical composition, and includes species of *L.* (*L.*) *biformis* Kirkpatrick, 1908 [61]; *L.* (*L.*) *bocagei* Ridley & Dendy, 1886 [62]; *L.* (*L.*) *apicalis* Ridley & Dendy, 1886 [62]; *L.* (*L.*) *brevis* Ridley & Dendy, 1886 [62]; *L.* (*L.*) *triverticillata* Alvarez, Bergquist & Battershill, 2002 [63]; *L.* (*L.*) *fiordensis* Alvarez, Bergquist & Battershill, 2002 [63]; *L.* (*L.*) *austini* Samaai, Gibbons & Kelly, 2006 [50]; *L.* (*L.*) *hamanni* Kelly, Reiswig & Samaai, 2016 [21]. Four species from the subgenus *Biannulata*, i.e., *L*. (*B*.) *purpurea* Carter, 1881 [64]; *L.* (*B*.) *wellingtonensis* Alvarez, Bergquist & Battershill, 2002 [63]; *L.* (*B*.) *kaikoura* Alvarez, Bergquist & Battershill, 2002 [63]; *L.* (*B*.) *citharistae* Vacelet, 1969 [65] (now accepted as *Latrunculia incertae sedis* and a possible species of *Sceptrella* by Kelly et al. 2016) [21] and only one species from the subgenus *Uniannulata* (*L*. (*U*.) *oparinae* Samaai & Krasokhin, 2002) [48] have reported chemistry. 

Numerous studies on the chemical composition of *Latrunculia* spp. have yielded diverse pyrroloiminoquinone-type alkaloids with the trivial name of discorhabdin, which was originally named after the discorhabd spicules [27,30]. Bispyrroloiminoquinones (tsitsikammamines) and simple pyrroloquinoline-type alkaloids (e.g., makaluvamines and batzellines), which have been proved to be biosynthetic precursors of tsitsikammamines and discorhabdins, were also reported from this genus [30,66,67]. Apart from the aforementioned alkaloids, sponges of the genus *Latrunculia* were reported to produce cyclodepsipeptides, the so-called callipeltins. This section of the review will emphasize the origin, chemistry and bioactivity of different types of (bis)pyrroloiminoquinone alkaloids and callipeltins deriving from the genus *Latrunculia*.

#### 3.1.1. Discorhabdin Alkaloids Obtained from the Genus *Latrunculia*

**Figure 2:**
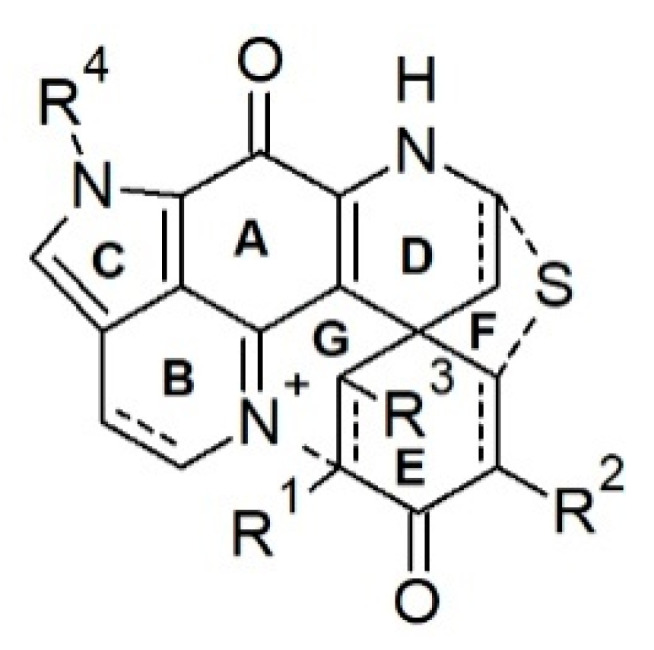
Skeleton of monomeric discorhabdin-type alkaloids.

Discorhabdins are pyrroloiminoquinone-type alkaloids possessing the characteristic tetracyclic pyrido[2,3-*h*]-pyrrolo-[4,3,2-*de*]quinoline core structure attached to an extra cyclohexanone ring via a carbocyclic spiro center C-6 (Figure 2), hence, forming a pentacyclic backbone [49,68]. Discorhabdins are reported from nature as monomers, dimers, and trimers, and mostly as trifluoroacetate (TFA) or formic acid (FA) salts with high or moderate polarity. Since the first report of discorhabdin C from an unidentified specimen of New Zealand *Latrunculia* [27], 35 monomeric and 11 dimeric/trimeric discorhabdins have been reported from the genus *Latrunculia*. 

Chemical diversity of the monomeric discorhabdin alkaloids stems from various substitutions on their pentacyclic backbone, the stereochemistry of C-1, and their enantiomeric properties. A sulfide linkage between C-5 and C-8 (e.g., in discorhabdins B and Q) [69,70] is common, allowing the formation of hexacyclic discorhabdins. Some discorhabdins bear a heptacyclic backbone (as found in discorhabdins L and D) via a further intramolecular ring closure formed by a direct bond between the N-18 imino function and C-2 (Figure 2) [71,72]. With the formation of the sulfide bridge between C-5 and C-8, the relative geometry between rings D and E is locked, hence, the configurational relativity between C-6 and C-8 is fixed, i.e., bonds C6/C5 and C-8/S are always *syn* (Figure 2) [73]. In the case of heptacyclic discorhabdins, an extra direct bond between C-2 and N-18 further locks rings B and E, allowing only one relative configuration at C-2 (Figure 2) [73]. Halogenation (mainly bromine and chlorine) is a common substitution in discorhabdins, mostly at positions C-2/C-4/C-14 (e.g., discorhabdins A and C, 1-hydroxy-14-bromodiscorhabdin V) [27,30,69,71]. Several enantiomers of known discorhabdins were also reported from nature (e.g., (+)/(−)-discorhabdin B, L, and I), further increasing the chemical diversity of discorhabdins [74,75]. According to the ring number of the discorhabdin backbone, monomeric discorhabdins can be classified into three categories, namely, the pentacyclic discorhabdin C series (e.g., discorhabdins E and G), hexacyclic discorhabdin B series (e.g., discorhabdins Q, R, and I) and heptacyclic discorhabdin D series (e.g., discorhabdins L, N, and H) [68]. 

Dimeric discorhabdins linked by a disulfide or sulfide bridge between two discorhabdin units (e.g., discorhabdin W, discorhabdin B dimer) have also been reported from sponges of the genus *Latrunculia* [76,77]. Several discorhabdin dimers bearing a novel C1-N13 direct bond between two discorhabdin units have been newly reported [78,79]. The Antarctic *Latrunculia biformis* Kirkpatrick, 1908 is the source of the very first trimeric discorhabdin containing the same C1-N13 bond [79]. Different configurations of C-1 in discorhabdin oligomers were observed [78,79]. 

Structure elucidation of discorhabdins has been hampered by the presence of heteroatoms (N, O, S, Br, and Cl) and high degree of unsaturation in such highly fused ring systems. Structures of some discorhabdins (e.g., (+)-discorhabdin A and discorhabdin C) have been secured by X-ray analysis. Hence, interpretations of NMR spectroscopic data with analogy to discorhabdins A and C have assisted the structure elucidation of discorhabdins [27]. NOE data analysis has been used to assign the relative configurations. Biogenetic considerations and [α]_D_ values have been used to propose the absolute configurations of discorhabdins, leading to inaccuracies. Since 2008, electronic circular dichroism (ECD) spectroscopy in conjunction with the computationally assisted density-functional theory (DFT)-based calculations have become a more efficient and reliable method to confirm the absolute configurations of discorhabdins [74,75]. 

#### Discorhabdin Monomers

**Figure 3:**
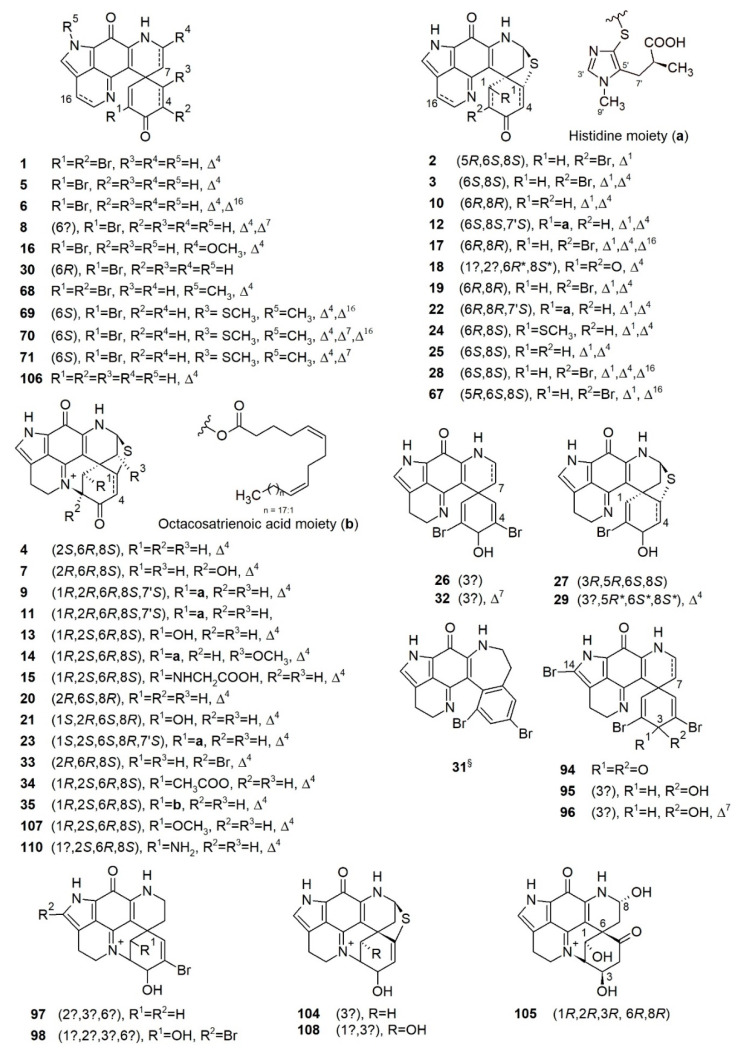
Discorhabdin monomers reported from latrunculid sponges. ?: Unassigned stereochemistry; *: Relative configuration; ^§^: Artifact.

The story of pyrroloiminoquinone-type alkaloids began in 1986 with the discovery of discorhabdin C (**1**) from an unidentified specimen of a New Zealand *Latrunculia* sp. [27]. Bioactivity-guided fractionation of this cytotoxic *Latrunculia* sp. extract led to the purification of a toxic pigment named discorhabdin C (**1**) (Figure 3) [27]. The structure of **1** was secured by X-ray diffraction analysis, but its NMR spectrum was left unassigned because of the highly fused ring systems [27]. Discorhabdin C (**1**) showed significant activity against mouse lymphocytic leukemia cell line L1210 with an ED_50_ value below 100 ng/mL. Since then, a series of chemically related analogs have been reported from different *Latrunculia* species collected from diverse sites around the world. Continuous chemical investigations on three sponges of the genus *Latrunculia* collected from several locations in New Zealand [69,71] led to the isolation of (+)-discorhabdin A (**2**) and (−)_546_-discorhabdin D (**4**) from a specimen identified as *L.* (*L.*) *brevis* Ridley & Dendy, 1886, while (+)-discorhabdin B (**3**) and discorhabdin C (**1**) were reported from an undescribed *Latrunculia* specimen (Figure 3) [69,71,80]. Compounds **2** and **3** bear a hexacyclic backbone including a sulfide bridge between C-5 and C-8, while **4** is a heptacyclic compound with an extra ring closure through the formation of a direct bond bound between C-2 and N-18 imino function. This is very rare for a small molecule with only 18 carbons [71]. Although (−)_546_-discorhabdin D (**4**) possesses three stereogenic centers, it lacked optical activity under the common sodium D light (589 nm), while negative optical rotation values were observed under 578 and 546 nm [71]. This study assigned the ^1^H and ^13^C-NMR data of discorhabdin C for the first time, and the chemical structures of **2** and **3** were established by the comparison of the spectral data with those of discorhabdin C (**1**) [69]. The structure elucidation of (−)_546_-discorhabdin D (**4**) (Figure 3) was also based on a comparison of its NMR data with compounds **1**–**3** [71]. Unlike discorhabdin C (**1**), discorhabdins A, B, and D (**2**–**4**) are chiral molecules, and the relative configurations of their stereocenters were assigned by means of a 1D NOE difference experiment. The absolute configurations of (+)-discorhabdin B (**3**) and (−)_546_-discorhabdin D (**4**) were confirmed in 2008 and 2010, respectively, via direct comparison of their experimental and DFT-calculated ECD spectra [74,75].

Discorhabdin C (**1**), A (**2**), and B (**3**) exhibited in vitro cytotoxicity against P388 leukemia cells with ED_50_ values of 0.03, 0.05, and 0.1 μg/mL, respectively [69,71], but none of them had any significant in vivo activity in the P388 leukemia system in mice [69]. Notably, discorhabdin D (**4**) that was less active against P388 leukemia cells (ED_50_ value 6 μg/mL) showed in vivo activity at a dose of 20 mg/kg with a treatment-to-control ratio (T/C) of 132% in the P388 leukemia system in mice [71]. Discorhabdins A (**2**) and C (**1**) exerted significant antimicrobial activity against *Escherichia coli*, *Bacillus subtilis* and *Candida albicans*, while discorhabdin B (**3**) was active against *E. coli* and *B. subtilis* [69]. However, the MIC/IC_50_ values of the compounds were not presented. 

In 1994, Copp et al. reported the isolation of a new 4-debromo derivative of discorhabdin C, named discorhabdin E (**5**), from a specimen identified as *L.* (*L.*) *bocagei* Ridley & Dendy, 1886 collected in the Auckland Islands, New Zealand (Figure 3) [80]. The elimination of the bromine atom generated a stereogenic center in **5**, which was purified as a racemic mixture of its TFA salt. Compound **5** showed significant in vitro cytotoxicity against P388 murine leukemia cell lines (IC_50_ value 206 ng/mL) and antimicrobial activity against *B. subtilis* and *E. coli* (IC_50_ values not reported) [80]. Notably, the structures of discorhabdins E (**5**), F (**6**), and 2-hydroxydiscorhabdin D (**7**) were first presented by Blunt et al. in a review article [81], where the authors reported these three compounds from an unidentified New Zealand *Latrunculia*. However, the NMR data of compounds **5**–**7** were not reported therein. 

Discovery of discorhabdin alkaloids from *Latrunculia* sp. continued with the report of (+)-discorhabdin G (**8**, Figure 3) together with discorhabdin C (**1**) from an Antarctic sponge identified as *L.* (*L.*) *apicalis* Ridley & Dendy, 1886 that showed significant antifeedant activity against the sea star *Perknaster fuscus*, the major predator of marine sponges in Antarctica [28]. This was also the first investigation of a *Latrunculia* specimen from a region other than New Zealand. (+)-Discorhabdin G (**8**) lacks the bromine atom at C-4 but bears the same pentacyclic core structure as discorhabdin C (**1**) with an additional double bond at C-7 [28]. (+)-Discorhabdin G (**8**) has a single stereogenic center (C-6) and exhibits a slightly positive [α]_D_ value. However, its absolute configuration remained undetermined [28]. (+)-Discorhabdin G (**8**) demonstrated significant antifeedant activity against *P. fuscus* and antimicrobial activity against two common water column microorganisms (the IDs of microorganisms not mentioned) isolated from seawater surrounding the sponge. Hence, compound **8** was hypothesized to be involved in the chemical defense of the sponge [28]. The following study by Furrow et al. (2003) revealed that compound **8** was mainly distributed in the outermost layer of the sponge (2 mm) that has the highest chance of encountering predators, which further emphasized the ecological significance of (+)-discorhabdin G [29].

Discorhabdins H–O (**9**–**16**) were isolated from another *Latrunculia* sp. from New Zealand and their structures were presented by Munro et al. at the 37th Annual ASP Meeting in 1996, then listed in a review paper in 2000 [49,82]. In the review paper, the structure of discorhabdin H was omitted. The structures of some compounds published in this review contain some errors, i.e., the inadvertent omission of the ∆^4^ olefin in discorhabdins L and N; and the omission of the sulfide bridge between C-5 and C-8 in discorhabdins I and K [30,49,75,82]. The 1D NMR, [α]_D,_ and CD data of all eight discorhabdins were inaccessible in the review, leading to some ambiguities in the nomenclature of newly discovered discorhabdins in the following studies.

In 1999, an orange solid was detected as the major component of cytotoxic extracts of three Australian sponges (*Zyzzya massalis* Dendy, 1922 [83] (accepted as *Zyzzya fuliginosa* Carter, 1879) [84], *Zyzzya.* sp., and *L.* (*B.*) *purpurea* Carter, 1881) and a Fijian sponge *Z. fuliginosa* Carter, 1879 [70,84]. The orange pigment was identified as 16,17-dehydrodiscorhabdin B with the trivial name (−)-discorhabdin Q (**17**) (Figure 3) because discorhabdin P has been reported from a Caribbean sponge of the genus *Batzella* (revised as *Strongylodesma*) [70,85]. (−)-Discorhabdin Q (**17**) bears the same core structure as discorhabdin B (**3**) and due to the olefinic bond at C-16, the tetracyclic pyrido[2,3-*h*]-pyrrolo-[4,3,2-*de*]quinoline portion of compound **17** is fully aromatic. (−)-Discorhabdin Q (**17**) exhibited a negative [α]_D_ value (−452.4). Based on biogenetic considerations, the absolute configuration of **17** was originally proposed to be 6*S*,8*S* [70], however, it was revised into 6*R*,8*R* in 2010, based on the assignment of a 6*S*,8*S* configuration to (+)-discorhabdin Q (**28**) obtained from a New Zealand-sourced *Latrunculia* sp. [75]. Compound **17** exhibited moderate cytotoxicity in the NCI 60 cell line antitumor screen (mean panel GI_50_ = 0.5 μg/mL) [70]. 

Ford and Capon (2000) reported another sulfur-containing compound (+)-discorhabdin R (**18**) from an Antarctic *Latrunculia* sp. (Figure 3) [86]. The gross structure of (+)-discorhabdin R (**18**) was elucidated as a debromo epoxy analog of the co-occurring discorhabdin B (**3**). The stereochemistry of all the other chiral centers except C-1 and C-2 were established via biosynthetic considerations and further confirmed by comparison of its NMR data with those of (+)-discorhabdin B (**3**). The orientation of the epoxy function to the rest of the molecule could not be confirmed due to the lack of decisive NOE correlations [86]. The bioactivity of the pure compounds was not tested in this study, but the EtOH extract of the source sponge exhibited antibacterial activity against both Gram-positive and Gram-negative bacteria (the IC_50_ or MIC (minimum inhibitory concentration) values were not mentioned in the publication).

Two new members of the discorhabdin family, namely (−)-discorhabdin I (**10**) and (−)_546_-discorhabdin L (**13**) were reported by Reyes et al. (2004) from the deep-sea Argentinian sponge *L.* (*L.*) *brevis* Ridley & Dendy, 1886 (Figure 3) [72]. At almost the same time, (−)-discorhabdin I (**10**) was also obtained from another South African latrunculid sponge (*Latrunculia bellae* Samaai & Kelly, 2003 (accepted as *Cyclacanthia bellae* Samaai & Kelly, 2003 [56])) and referred to as discorhabdin G* [30]. Discorhabdin G*/I (**10**) is a debromo analogue of discorhabdin B (**3**) and exhibited a negative [α]_D_ value. Discorhabdin L (**13**) is a heptacyclic compound bearing the same skeleton as discorhabdin D (**4**) with the only difference being the presence of a hydroxy group at C-1 in **13**. Although discorhabdin L (**13**) possesses four stereogenic centers, it was optically inactive at sodium D light (589 nm) but showed negative [α]_D_ values under 578 and 546 nm, a phenomenon previously observed with discorhabdin D (**4**) [72,74]. The absolute configurations of **10** and **13** were confirmed only in 2008 by ECD spectroscopy [74]. Compounds **10** and **13** were tested against 14 different cancer cell lines, where they showed the best potency against the colon cancer cell line HT-29 with GI_50_ values of 0.35 and 0.12 μM, respectively [72]. 

Discorhabdins I (**10**), B (**19**), D (**20**), and L (**21**) were reisolated from a *Latrunculia* sp. collected from Milford Sound, New Zealand [76]. In a subsequent study, Grkovic et al. (2008) assigned the optical rotation and absolute configurations of these compounds as (−)-discorhabdin G*/I (**10**), (−)-discorhabdin B (**19**), (+)_546_-discorhabdin D (**20**) and (+)_546_-discorhabdin L (**21**) [74]. All four compounds exhibited promising in vitro cytotoxicity against P388 lymphocytic leukemia cells, with **19** being the most potent (GI_50_ value 0.087 μM). Compounds **10**, **20**, and **21** showed relatively weak activity, with IC_50_ values ranging from 0.51 to 1.6 μM [76]. 

Between 2008 and 2010, the Copp research group explored the chemical composition of *Latrunculia* sponges collected from different locations in New Zealand [74,75,87]. They reported three new monomeric discorhabdin analogs, i.e., (−)-discorhabdin K_2_ (**22**), (+)-discorhabdin H_2_ (**23**), and (+)-1-thiomethyldiscorhabdin G*/I (**24**); three new enantiomers of known discorhabdins, i.e., (−)-discorhabdins B (**19**), (+)_546_-discorhabdin L (**21**), and (+)-discorhabdin-G*/I (**25**); and numerous known discorhabdins, some of which have never been reported, from the genus *Latrunculia*, e.g., (−)-3-dihydrodiscorhabdin C (**26**, originated from *Tsitsikamma pedunculata* Samaai & Kelly, 2003 [56]) and (+)-3-dihydrodiscorhabdin A (**27**, originated from *Higginsia* sp.) (Table 1; Figure 3) [30,74,75,87]. The absolute configurations of several known discorhabdins, e.g., (−)-discorhabdin H (**9**), (+)-discorhabdin K (**12**), (−)_546_-discorhabdin L (**13**) were also confirmed in this series of studies [74,75,87]. Both discorhabdins H (**9**), H_2_ (**23**) bear a thiomethylhistidine fragment connected to a discorhabdin l-type core structure at C-1 via a sulfide bridge (Figure 3). The absolute configuration of the thiomethylhistidine substituent in (−)-discorhabdin H (**9**) has been established as 7’*S* by Antunes et al. (2004) through degradative techniques where compound **9** was purified from a South African sponge *Strongylodesma algoaensis* Samaai & Kelly, 2003 [30,56], but the absolute configuration of the stereocenters in the core discorhabdin structure was not assigned [30]. Grkovic et al. (2010) confirmed the absolute configuration of (−)-discorhabdin H (**9**) as 1*R*,2*R*,6*R*,8*S*,7′*S* by comparing its experimental ECD spectrum with the model compound (−)_546_-(1*R*,2*R*,6*R*,8*S*)-discorhabdin L (**13**) [75]. Compared to (−)-discorhabdin H (**9**), (+)-discorhabdin H_2_ (**23**) exhibited essentially equal and opposite experimental ECD spectra, and hence established the absolute configuration of (+)-discorhabdin H_2_ (**23**) as 1*S*,2*S*,6*S*,8*R*,7′*S*. The authors concluded that the induced circular dichroism properties of (−)-discorhabdin H (**9**) and (+)-discorhabdin H_2_ (**23**) were due to the core discorhabdin structure only [75]. The absolute configurations of (+)-discorhabdin K (**12**) and (−)-discorhabdin K2 (**22**) were established by the strategy used for discorhabdins H (**9**) and H2 (**23**) [75]. 

DFT calculations of ECD spectra were used, for the first time, in stereochemical assignments of discorhabdin analogs allowing the stereochemical revision of (+)-3-dihydrodiscorhabdin A (**27**) (from 3*S*,5*R*,6*S*,8*S* to 3*R*,5*R*,6*S*,8*S*), the establishment of absolute configurations for (−)_546_-discorhabdin D (**4**), (+)-2-hydroxydiscorhabdin D (**7**), (−)-discorhabdin H (**9**), (+)-discorhabdin K (**12**), (−)-discorhabdin N (**15**), (−)-discorhabdin K2 (**22**), (+)-discorhabdin H_2_ (**23**), and (+)-discorhabdin Q (**28**) [74,75,87]. Since then, DFT-based ECD calculations have become one of the most reliable and effective strategies to confirm the absolute configuration of discorhabdin-type alkaloids. Six sponges of the genus *Latrunculia* collected from four different sites of New Zealand (Table 1) were studied to investigate the relationships between sponge phenotypes and their production of discorhabdin enantiomers [74,75,87]. The results revealed that Milford/Doubtful Sound-sourced sponges of the genus *Latrunculia* produced discorhabdins with an α-orientated C-6/C-5 bond when their skeleton was drawn as Figure 3, e.g., (−)-discorhabdin B (**19**), (−)-discorhabdin-G*/I (**10**), and (+)_546_-discorhabdin L (**21**) (Table 1), while Wellington Harbor/Kaikoura Coast-sourced sponges of the genus *Latrunculia* yielded the corresponding enantiomeric discorhabdins, i.e., (+)-discorhabdin B (**3**), (+)-discorhabdin G/I (**25**), and (−)_546_-discorhabdin L (**13**) (Table 1) [74,75,87]. This series of studies highlight the enantiomeric specificity in the biosynthesis of thioether-containing discorhabdins, but the relationship with the collection sites is still not clear due to the limited dataset [74,75,87]. 

The new compounds (−)-discorhabdin K_2_ (**22**), (+)-discorhabdin H_2_ (**23**), and (+)-1-thiomethyldiscorhabdin G*/I (**24**); enantiomeric pairs of known discorhabdins, i.e., (−)-discorhabdin B (**19**), (+)_546_-discorhabdin L (**21**), and (+)-discorhabdin-G*/I (**25**) together with compounds (−)-discorhabdin-H (**9**), (+)-discorhabdin-K (**12**), (+)-3-dihydrodiscorhabdin A (**27**) and its semi-synthetic 3-epimer (+)-(3*S*)-3-dihydrodiscorhabdin A were tested on P388 murine leukemia cell lines to investigate their cytotoxicity and structure–activity relationships (SARs). The enantiomeric pairs of discorhabdins B ((+)/(−)-B, **3**/**19**), I ((+)/(−)-I, **25**/**10**), and L ((+)/(−)-L, **21**/**13**)) displayed similar cytotoxicity, with IC_50_ values ranging from 0.17 to 1.08 μM, while (−)-discorhabdin-H (**9**), (+)-discorhabdin-K (**12**), (−)-discorhabdin K_2_ (**22**) and (+)-discorhabdin H_2_ (**23**) were weaker with IC_50_ values higher than 8.2 μM [74,75,87], suggesting that the C-1 substituation is detrimental to the anticancer activity of discorhabdins.

Since 2010, several *Latrunculia* sponges from the North Pacific Ocean have been studied for their chemical composition, yielding many new discorhabdin alkaloids [32,66,88,89,90]. An unidentified specimen of *Latrunculia* from the Aleutian Islands (Bering Sea, −230 m depth), now known as *L.* (*L.*) *hamanni* Kelly, Reiswig & Samaai, 2016, afforded the new alkaloids, 3-dihydrodiscorhabdin B (**29**) and (+)-discorhabdin Y (**30**), which is the 4-debromo derivative of discorhabdin C, and six known metabolites (+)-discorhabdin A (**2**), discorhabdin C (**1**), E (**5**), and L (**13**), 3-dihydrodiscorhabdin C (**26**), and the benzene derivative of discorhabdin C (**31**) (Figure 3) [32]. The latter compound (**31**), which was synthesized by Copp et al. (1994) via chemical modification of discorhabdin C was deemed to be an artifact of the isolation process [32,80]. Compound **29**, 3-dihydrodiscorhabdin B, was unstable and decomposed rapidly before [α]_D_ measurements. Discorhabdin Y (**30**) showed good stability and its absolute configuration was confirmed as (+)-(6*R*)-discorhabdin Y (**30**) based on its [α]_D_ data and computationally-assisted ECD calculations [32]. This study investigated, for the first time, the in vitro antiviral and antimalarial activity of discorhabdin C (**1**), (+)-discorhabdin A (**2**), and 3-dihydrodiscorhabdin C (**26**). All compounds were active in the HCV (hepatitis C virus) Huh-7 replicon assay with EC_90_ values below 10 μM [32]. Compounds **1**, **2,** and **26** displayed antiplasmodial activity against both the chloroquine-susceptible *P. falciparum* D6 clone (IC_50_s 2.8, 0.053 and 0.17 μM, respectively), and the chloroquine-resistant W2 clone (IC_50_s 2.0, 0.053, and 0.13 μM, respectively) [32]. When evaluated for general toxicity toward the monkey kidney fibroblasts (Vero) cells, (+)-discorhabdin A (**2**), showed the highest selectivity index (SI = 130) followed by 3-dihydrodiscorhabdin C (**26**) (SI = 58) and discorhabdin C (**1**) (SI = 1). During in vivo studies of compounds **2** and **26** in a *P. berghei* mouse malaria model at a dose of 10 mg/kg, animals treated with 3-dihydrodiscorhabdin C (**26**) died due to toxicity on day 4 before the examination for parasitemia, whereas the animals treated with (+)-discorhabdin A (**2**) had to be euthanized because of significant weight loss (>25%) and severe signs of toxicosis despite the significant (approx. 50%) suppression of parasitemia [32]. In addition, discorhabdin C (**1**), (+)-discorhabdin A (**2**), and 3-dihydrodiscorhabdin C (**26**) also showed significant antimicrobial activity against MRSA, *Mycobacterium intracellulare* and *M. tuberculosis* with IC_50_ values ranging from 0.13 to 13 μM [32].

*Latrunculia* (*U.*) *oparinae* Samaai & Krasokhin, 2002, collected near the shores of Kuril Islands, Sea of Okhotsk (Russia), afforded the ethanol solvate of discorhabdin A (**2**) [88] with an opposite [α]_D_ value (−449) to the Okinawan *Strongylodesma* sp. sourced discorhabdin A ([α]_D_ +400) [69,91], although they have the same absolute configuration. This was due to the unique crystal structure of EtOH solvated discorhabdin A, whose [α]_D_ value turned positive after three days of storage in MeOH [88]. This compound showed potent cytotoxicity against murine Ehrlich carcinoma cells (ED_50_ = 0.055 μg/mL). 

Hypoxia-inducible factor 1α (HIF-1α) is a subunit of hypoxia-inducible factors that is overexpressed in many human tumors [92]. Inhibition of HIF-1α by disrupting its binding to the transcriptional coactivator protein p300 is a validated target in cancer drug discovery [93,94,95]. (+)-Discorhabdin B (**3**), 3-dihydrodiscorhabdin C (**26**), and (+)-discorhabdin G*/I (**25**) (Figure 3) [77] isolated from an Australian *Latrunculia* sp. as well as five previously isolated discorhabdins namely, (+)-discorhabdin A (**2**), (−)_546_-discorhabdin D (**4**), (−)-discorhabdin-H (**9**), (−)_546_-discorhabdin L (**13**), and (−)-discorhabdin N (**15**) from a New Zealand-sourced *Latrunculia* sp. were tested for their inhibitory potential for HIF-1α/p300 interaction [77]. Compounds **3**, **13**, **9**, and **26** were the most potent HIF-1α/p300 inhibitors, with IC_50_ values ranging from 0.73 to 35.2 μM. All four compounds demonstrated in vitro cytotoxicity under normoxic conditions [30], therefore the authors concluded that oxygen could mediate the cytotoxicity of pyrroloiminoquinone alkaloids [77]. In the following in vivo anticancer activity study on (−)-discorhabdin-H (**9**) and (−)_546_-discorhabdin L (**13**) in a prostate cancer (LNCaP) xenograft model, only compound **13** showed significant inhibition of LNCaP tumor growth and no reduction in body weight of LNCaP-bearing mice after four weeks of treatment at a dose of 5 mg/kg [96]. This study shed some light on the anticancer mechanism of discorhabdins. 

Botic et al. [33] studied the methanolic extracts of two Antarctic deep-water specimens, identified as *L.* (*L.*) *biformis* Kirkpatrick, 1908 and *L.* (*L.*) *bocagei* Ridley & Dendy, 1886, to obtain (+)-discorhabdin G (**8**) and (−)-3-dihydro-7,8-dehydrodiscorhabdin C (**32**) (from *L.* (*L.*) *biformis* Kirkpatrick, 1908) and (+)-discorhabdin B (**3**) and (−)_546_-discorhabdin L (**13**) (from *L.* (*L.*) *bocagei* Ridley & Dendy, 1886) (Figure 3) [33]. (+)-Discorhabdin G (**8**) appeared as the most potent competitive reversible inhibitor of electric eel acetylcholinesterase (EeAChE) and butyrylcholinesterase (BChE) enzymes, whereas compound **3** was the most potent competitive reversible inhibitor of human acetylcholinesterase (hAChE). In docking studies, (+)-discorhabdin G (**8**) showed the lowest value of binding energy and the highest number of hydrophobic interactions with EeAChE. Similarly, the observed activity of (+)-discorhabdin B (**3**) against hAChE was supported by a series of hydrophobic interactions and three relevant H-bonds in the complex formed with the enzyme. Furthermore, (+)-discorhabdin G (**8**) did not show undesirable side effects as EeAChE and BChE inhibitors, pointing out the potential of the discorhabdin scaffold as a new class of potent cholinesterase inhibitors with minimal side effects [33]. 

Our research group investigated the Antarctic deep-sea sponge *L.* (*L.*) *biformis* Kirkpatrick, 1908 that exhibited activity against multiple cancer cell lines. Molecular networking (MN)-based untargeted metabolomics analyses of the methanol soluble portion of the crude extract was found to be dominated by polar, monomeric discorhabdins (e.g., known discorhabdins A, D, G, and L) and some putatively new derivatives. Targeted isolation studies furnished three new compounds, (−)-2-bromodiscorhabdin D (**33**), (−)-1-acetyldiscorhabdin L (**34**), and (+)-1-octacosatrienoyldiscorhabdin L (**35**) along with the known discorhabdins, namely, (−)-discorhabdins L (**13**), (+)-discorhabdin Q (**28**) and (+)-discorhabdin A (**2**) (Figure 3) [73]. Their absolute configurations were assigned by comparison of the experimental CD spectra with that of the standard compound (−)_546_-discorhabdin L (**13**). This study reported the first discorhabdin alkyl esters (**34** and **35**) from nature. Compound **35** is an ester of (−)_546_-discorhabdin L (**13**) with an unbranched octacosatrienoic acid (C28:3) that has never been reported from any *Latrunculia* species before [73]. Upon testing against HCT-116 colon cancer cells in vitro, (−)_546_-discorhabdins L (**13**), (−)-1-acetyldiscorhabdin L (**34**), and (+)-1-octacosatrienoyldiscorhabdin L (**35**) demonstrated activities with IC_50_ values of 0.94 μM (**13**), 2.71 μM (**34**), and 34.0 μM (**35**), respectively, suggesting that a substitution at C-1, especially the presence of a long chain fatty acid, was detrimental to the anticancer activity of discorhabdins [73]. A molecular modeling study using two known anticancer targets (topoisomerase I/II, indoleamine 2,3-dioxygenase (IDO1)) suggested these proteins as potential anticancer targets of discorhabdins. The partly aromatic pyrroloiminoquinone core was proven to be necessary for the cytotoxicity of discorhabdins, which formed an aromatic π-π stacking interaction with the targets [73]. Compound **35** did not show plausible binding modes against any of these target proteins, owing to the large side chain at C-1 [73]. Both (−)_546_-discorhabdins L (**13**) and its enantiomer (**21**) yielded plausible binding affinity instead against all the targeted proteins, indicating that discorhabdins are non-specific ligands and that the stereochemistry is not crucial for their anticancer action [73].

Discorhabdin C (**1**) has been reisolated from the purple-colored fractions of the New Zealand sponge *L.* (*L.*) *triverticillata* Alvarez, Bergquist & Battershill, 2002 [78]. Discorhabdin C (**1**) and eight known monomeric discorhabdins, (+)-discorhabdin B (**3**), 3-dihydrodiscorhabdin C (**26**), (−)_546_-discorhabdin D (**4**), (−)-discorhabdin H (**9**), (+)-discorhabdin H_2_ (**23**), (−)_546_-discorhabdin L (**13**), (−)-discorhabdin Q (**17**), and discorhabdin U (**71**, originating from *Strongylodesma purpurea* Samaai & Kelly, 2009 [57]) were evaluated for their antiparasitic activities and general toxicity on L6 rat myoblast cell line [78]. All tested compounds showed some degree of antimalarial activity towards *Plasmodium falciparum* K1 dual drug-resistant strain (*Pf* K1), with (−)_546_-discorhabdin L (**13**) being the most potent with an IC_50_ value of 30 nM and a SI of 19. (−)_546_-Discorhabdin L (**13**) inhibited the growth of the African trypanosome *Trypanosoma brucei rhodesiense* with an IC_50_ value of 0.4 μM [78].

#### Discorhabdin Oligomers 

**Figure 4:**
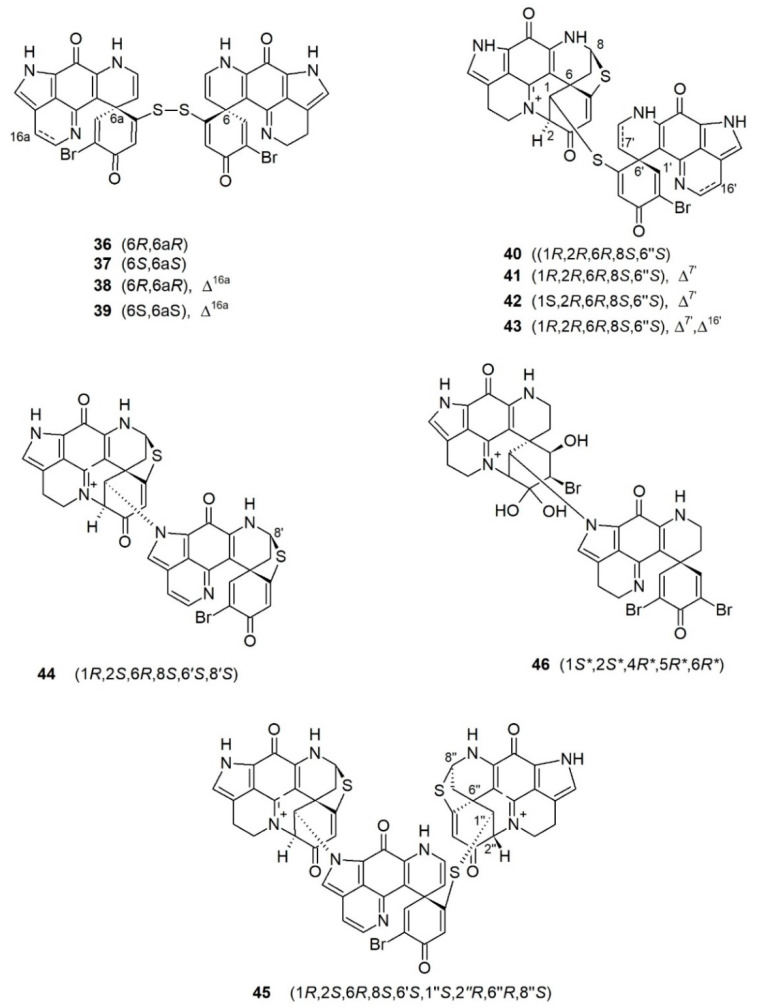
Discorhabdin oligomers reported from latrunculid sponges. *: Relative configuration.

Compared to monomeric discorhabdins, dimeric and trimeric discorhabdins are relatively rare in nature. Ten dimeric discorhabdins and only one trimeric discorhabdin have so far been reported. Depending on the atom(s) linking the monomeric units, discorhabdin oligomers can be divided into three categories: (a) those with a disulfide bridge as observed in (+)/(−)-discorhabdins W (**36**/**37**) and (+)/(−)-16a,17a-dehydrodiscorhabdins W (**38**/**39**); (b) those with a sulfide bridge as observed in discorhabdin B dimers (**40**–**43**); and (c) those with a direct C-1/N-13 bond as observed in didiscorhabdin, tridiscorhabdin, and discorhabdin C dimer (**44**–**46**) (Figure 4). 

(+)-Discorhabdin W (**36**) is the very first dimeric discorhabdin sourced from a New Zealand *Latrunculia* sp. collected from Milford Sound (Figure 4) [76]. It is a symmetrical compound with two identical discorhabdin B-type units connected through a disulfide bridge as established by NMR spectroscopy and chemical means [76]. Chemical reduction of **36** with dithiothreitol (DTT) yielded the co-occurring metabolite discorhabdin B, while irradiation of discorhabdin B under sunlight further promoted its dimerization into (+)-discorhabdin W (**36**) [76]. Hence, the absolute configuration of (+)-discorhabdin W (**36**) was considered to be the same as the co-occurring metabolites, e.g., (−)-discorhabdin G*/I (**10**), (−)-discorhabdin B (**19**), (+)_546_-discorhabdin D (**20**), and (+)_546_-discorhabdin L (**21**) (Figure 4). Both (+)-discorhabdin W (**36**) and (−)-discorhabdin B (**19**) exert equal anticancer potency against P388 lymphocytic leukemia cells (IC_50_ values 0.084 and 0.087 μM, respectively) [76], while compounds **10**, **20**, and **21** had lower activity with IC_50_ values of 1.6, 0.51 and 1.1 μM, respectively [76].

Grkovic et al. reported two pairs of enantiomeric discorhabdin dimers, (+)/(−)-discorhabdin W (**36** and **37**) and (+)/(−)-16a,17a-dehydrodiscorhabdin W (**38** and **39**) (Figure 4) from sponges of the genus *Latrunculia* collected from different sites in New Zealand [74,75,87]. The dimers displayed potent cytotoxicity against P388 murine leukemia cell lines in a similar magnitude to the “parent” monomeric discorhabdins with IC_50_ values of 0.10 and 0.13 μM for (+) and (−)-discorhabdin W (**36** and **37**), respectively, and 0.45 μM for both enantiomeric pairs of 16a,17a-dehydrodiscorhabdin W (**38** and **39**) [74,87]. 

Dimeric discorhabdin B (**40**) and (−)-discorhabdin W (**37**) were isolated from an Australian *Latrunculia* sp. that showed activity in the HIF-1α/p300 interaction in a cell-free protein−protein assay (Figure 4) [77]. Compound **40** was deemed to have the same structure as the previously reported artifact (−)-discorhabdin B dimer (**41**), which formed after long-term storage of discorhabdin B [97]. However, from the observed NMR data and the structure shown in the article, compound **40** should be a new 7’,8’-dihydro derivative of compound **41** [77,97]. Compound **40** inhibited the HIF-1α/p300 interaction with an IC_50_ value of 2.4 μM, and compound **37** was inactive (IC_50_ value > 100 μM) [77]. However, **40** demonstrated no cytotoxicity against HCT-116 and LNCaP cell lines under hypoxic conditions [77].

Molecular networking-based metabolomics strategy revealed the presence of several putatively new dimeric and trimeric discorhabdins in the *n*-hexane subextract of the Antarctic deep-sea sponge *L.* (*L.*) *biformis* Kirkpatrick, 1908 [79,98]. Targeted isolation efforts yielded three new dimeric discorhabdins, namely, (−)-1-*epi*-discorhabdin B dimer (**42**), (−)-16′,17′-dehydrodiscorhabdin B dimer (**43**), and didiscorhabdin (**44**), the known (−)-discorhabdin B dimer (**41**) and a trimeric molecule tridiscorhabdin (**45**) (Figure 4) [79,98]. Their absolute configurations were assigned by comparison of the experimental and the DFT-calculated ECD spectra. The dimeric discorhabdins comprised a discorhabdin L- and a discorhabdin Q-type unit, while tridiscorhabdin (**45**) contained two discorhabdin L- and a discorhabdin Q-type units. Compounds **41**–**43** bear a sulfide linkage between two discorhabdin monomers, whereas didiscorhabdin (**44**) and tridiscorhabdin (**45**) represent the first examples of discorhabdin oligomers linked with a direct C1–N13 bond [79,98]. Furthermore, tridiscorhabdin (**45**) is the first trimeric discorhabdin oligomer ever reported from nature. Compounds **41**, **42**, and **45** all exhibited strong in vitro cytotoxicity against human colon cancer cell line HCT-116, but were also toxic to the noncancerous human keratinocyte cell line HaCaT with IC_50_ values of 0.16 and 0.56 μM (**41)**, 2.01 and 4.69 μM (**42**) and 0.31 and 0.94 μM (**45**), respectively, indicating their low selectivity.

Shortly after the report of didiscorhabdin (**44**) and tridiscorhabdin (**45**) in 2020, the Copp group have published another C1-N13-linked discorhabdin C dimer (**46**) from the New Zealand sponge *L.* (*L*.) *trivetricillata* Alvarez, Bergquist & Battershill, 2002 (Figure 4) [78]. Discorhabdin C dimer (**46**) is the first tribrominated discorhabdin which is composed of two discorhabdin C-type units. Compound **46** was isolated as a racemate, because no specific rotation nor circular dichroism Cotton effects were observed at any wavelengths. Compounds **46** and **41** showed antimalarial (*Pf* K1 clone, IC_50_ values 6.4 and 0.08 μM, respectively) and antitrypanosomal activity against *T. brucei rhodesiense* (IC_50_ values 0.71 and 0.33 μM, respectively) [78]. Discorhabdin B dimer (**41**) was the least toxic against non-cancerous L6 rat skeletal myoblast cell lines (IC_50_ = 41 μM) with a very high selectivity (SI = 510), while compound **46** showed no selectivity (IC_50_ = 2.1 μM against L6 cells, SI = 0.3) [78].

#### 3.1.2. Tsitsikammamines 

**Figure 5:**
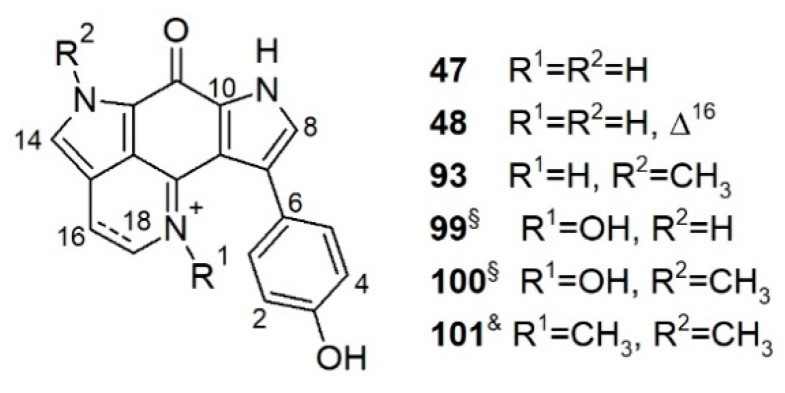
Tsitsikammamines reported from latrunculid sponges. ^§^: Artifact; ^&^: Reported from *Zyzzya* sp.

Tsitsikammamines are a small bispyrroloiminoquinone subclass that bear a very similar backbone and close biosynthetic relationships with discorhabdins [82]. Tsitsikammamines possess the same pyrrolo[4,3,2-*de*]quinoline ring system as discorhabdins and a characteristic C-6 *p*-oxybenzyl group that differs from discorhabdins (Figure 5). This chemical subfamily (Figure 5) is represented by only six compounds, i.e., tsitsikammamines A, B, and C, 16,17-dehydrotsitsikammamine A, and the *N*-oxime analogs of tsitsikammamines A and B [30,67,99,100].

Tsitsikammamines are rare alkaloids, and so far, have only been reported from another latrunculid, genus *Tsitsikamma*, and genus *Zyzzya* de Laubenfels, 1936 (order Poecilosclerida, family Acarnidae Dendy, 1922) [67]. However, a comprehensive MN-based untargeted metabolome study carried out by our research group on Antarctic *L.* (*L.*) *biformis* Kirkpatrick, 1908 indicated the presence of a small tsitsikammamine cluster (together with many discorhabdin analogs) in this sponge extract. Mass spectrometry-based targeted isolation of this cluster afforded tsitsikammamine A (**47**), and its new derivative 16,17-dehydrotsitsikammamine A (**48**) (Figure 5) [67]. This is the very first report of tsitsikammamines from the genus *Latrunculia*. Molecular docking of **47** and **48** into the active sites of topoisomerase-I and II as well as indoleamine 2,3-dioxygenase (IDO1) enzymes revealed plausible binding modes of both compounds. The aromatic pyrroloiminoquinone core structure that formed a π-π interaction with the target proteins was found to be essential for the cytotoxicity of tsitsikammamines **47** and **48**. Docking study against IDO1 revealed that an olefinic bond at ∆^16^ can diminish the in vitro cytotoxicity of tsitsikammamines [67]. 

#### 3.1.3. Rearranged Pyrroloiminoquinone-Type Alkaloids 

**Figure 6:**
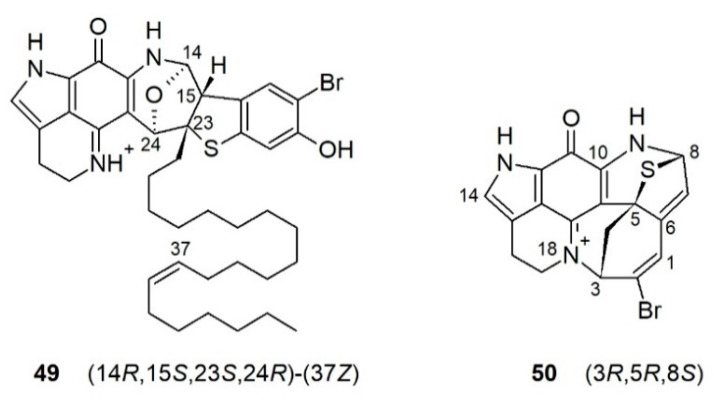
Chemical structure of atkamine and aleutianamine.

The Hamann group reported an unusual alkaloid atkamine (**49**) (Figure 6) from a deep-water sponge, *L*. (*L*.) *austini* Samaai, Gibbons & Kelly, 2006, from the Aleutian Islands, Alaska [89]. Atkamine (**49**) has a unique rearranged pyrroloiminoquinone scaffold with a highly fused ring system joined with several heteroatoms and a mono-unsaturated alkyl chain of 20 carbons. The position of the double bond was confirmed by fragmentation using an olefin metathesis. The absolute configuration of atkamine (**49**) was assigned using ECD spectroscopy combined with DFT calculations. The discovery of atkamine (**49**) prompted a re-collection of another sponge specimen *L.* (*L.*) *hamanni* Kelly, Reiswig & Samaai, 2016,**** during an NOAA (National Oceanic and Atmospheric Administration) deep ocean survey in Alaska [90]. MN-based targeted isolation studies afforded a new alkaloid, aleutianamine (**50**) (Figure 6) that possesses a highly strained heptacyclic ring system where a seven-membered azaheterocycle is formed via the rearrangement of rings D and F in a typical discorhabdin skeleton, plus a characteristic thioether bridge between C-5 and C-8 together with a direct bond between N-18 and C-3, which is different from the other heptacyclic discorhabdins (Figure 6) [90]. The structure of aleutianamine (**50**) was confirmed by DFT calculations of its 1D NMR and ECD data, plus the calculation of interproton distances between all non-exchangeable protons that were compared to the experimental ROE correlations. Aleutianamine (**50**) exhibited significant in vitro cytotoxicity toward pancreatic (PANC-1) and colon cancer (HCT-116) cell lines (IC_50_ values 25 nM and 1μM, respectively). 

#### 3.1.4. Citharoxazole, Batzelline, and Makaluvamine

**Figure 7:**
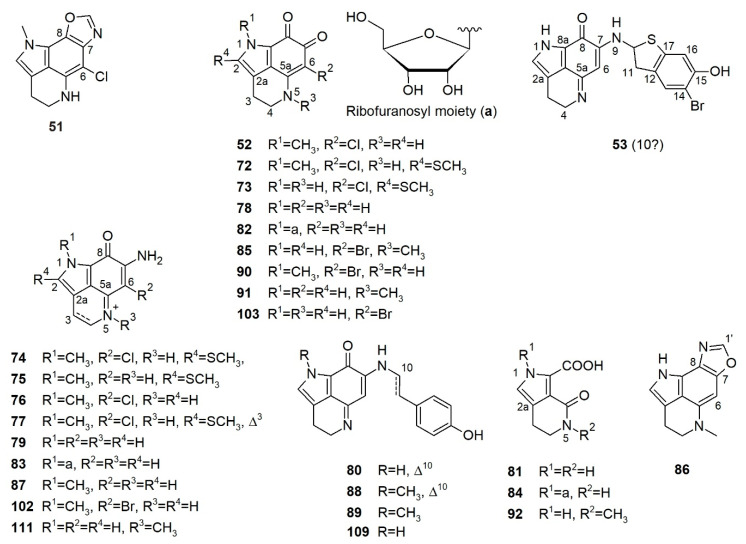
Citharoxazole, batzelline, and makaluvamine reported from the genus *Latrunculia*. ?: Unassigned stereochemistry.

Some simple cyclized pyrroloiminoquinone-related alkaloids were also reported from latrunculid sponges, e.g., makaluvamines, makaluvone, citharoxazole, makaluvic acids, batzellines, isobatzellines, and glycosylated analogues (Figure 7), out of which three compounds were reported from *Latrunculia* sp., namely, citharoxazole (**51**), batzelline C (**52**), and (−)-makaluvamine F (**53**) (Figure 7). All these alkaloids bear the same aminoquinonline ring system as discorhabdins and tsitsikammamines (Figure 7). These simple cyclic alkaloids have been proposed as important precursors in the biosynthesis of more complex pyrroloiminoquinones such as discorhabdins and tsitsikammamines [82]. 

In 2011, a French group investigated the metabolome of a sponge identified as *L.* (*B.*) *citharistae* Vacelet, 1969 (now accepted as *Latrunculia incertae sedis* and a possible species of *Sceptrella* by Kelly et al. 2016) [21], the only described latrunculid sponge from the Mediterranean Sea (103 m depth) [66], and reported a new discorhabdin-related alkaloid, citharoxazole (**51**), and the known compound batzelline C (**52**) (Figure 7). Both **51** and **52** bear a chlorine atom at C-6, and **51** is a highly unsaturated compound possessing an unusual oxazole ring attached to C-7 and C-8. The positions of the nitrogen and oxygen atoms of the oxazole ring were established through the coupling constant differences between ^3^*J*_H1’-C7_ and ^3^*J*_H1’-C8_ [66]. No bioactivity was reported for compounds **51** or **52**.

Makaluvamine F, a sulfur-containing alkaloid originally reported from a Fijian sponge, *Zyzzya massalis* Dendy, 1922 (now accepted as *Zyzzya fuliginosa* Carter, 1879 [37]) bears the typical pyrrolo[4,3,2-*de*]quinoline ring system (Figure 7) similar to discorhabdins and tsitsikammamines. It is regarded as the direct biosynthetic precursor of sulfur-containing discorhabdins (see Section 5) [82]. (−)-Makaluvamine F (**53**) was reported only from a New Zealand sponge identified as *Latrunculia* sp. in 2016 and shown to be a promising inhibitor of the HIF-1α/p300 protein–protein interactions (IC_50_ value 8.3 μM). It also exhibits moderate in vitro cytotoxicity against the HCT-116 cell line (IC_50_ value 7.2 μM) under hypoxic conditions [77].

#### 3.1.5. Callipeltins

**Figure 8:**
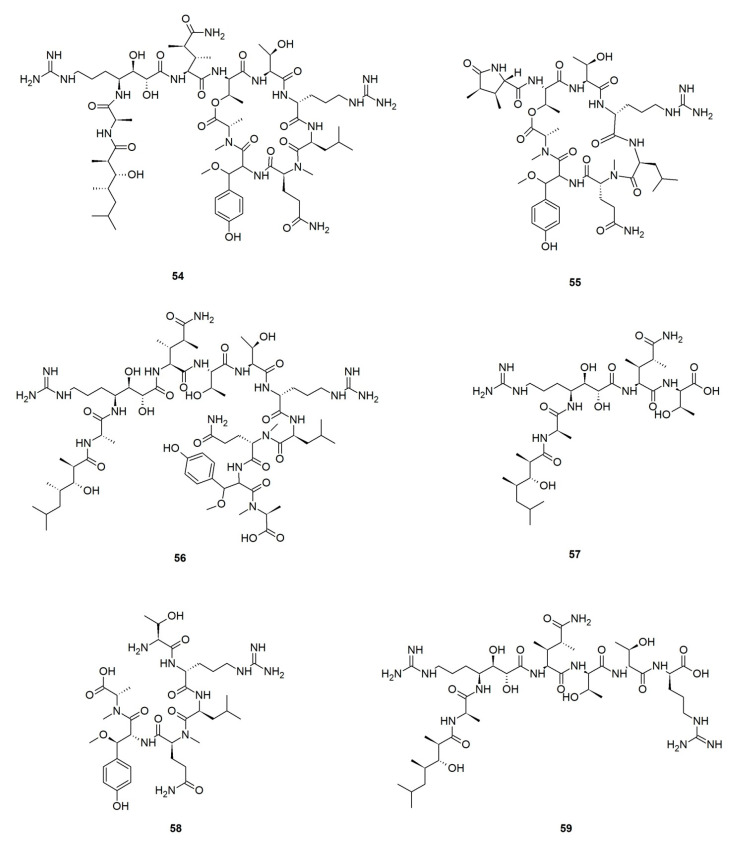

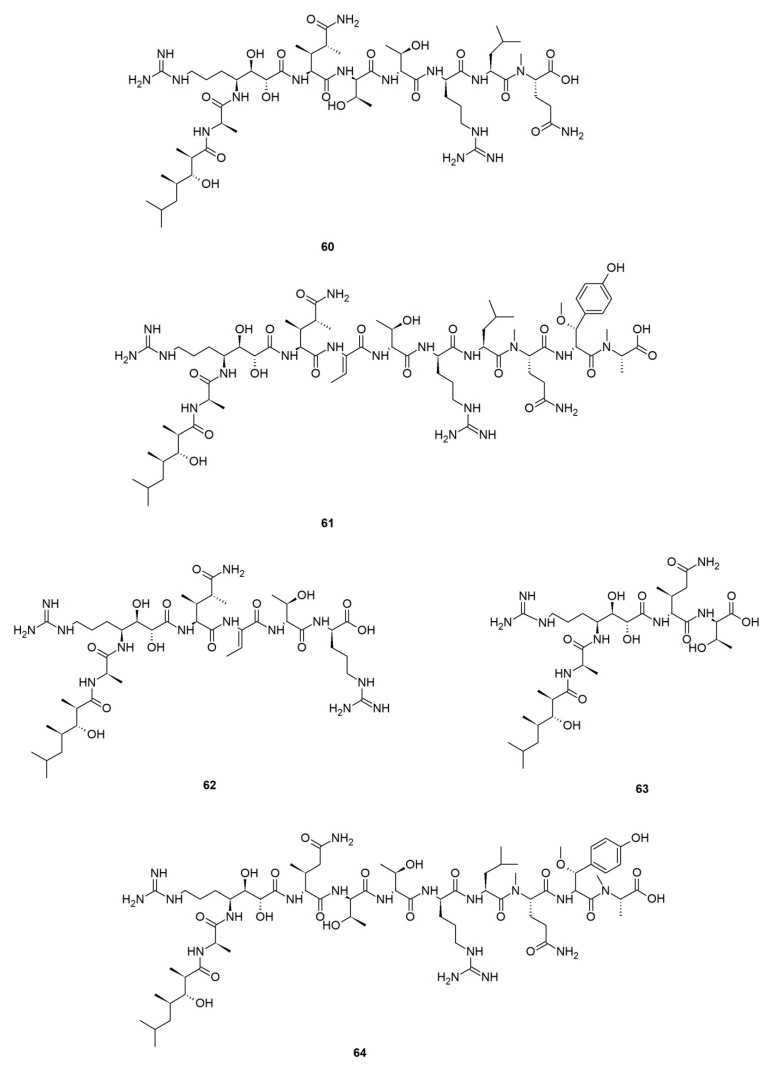

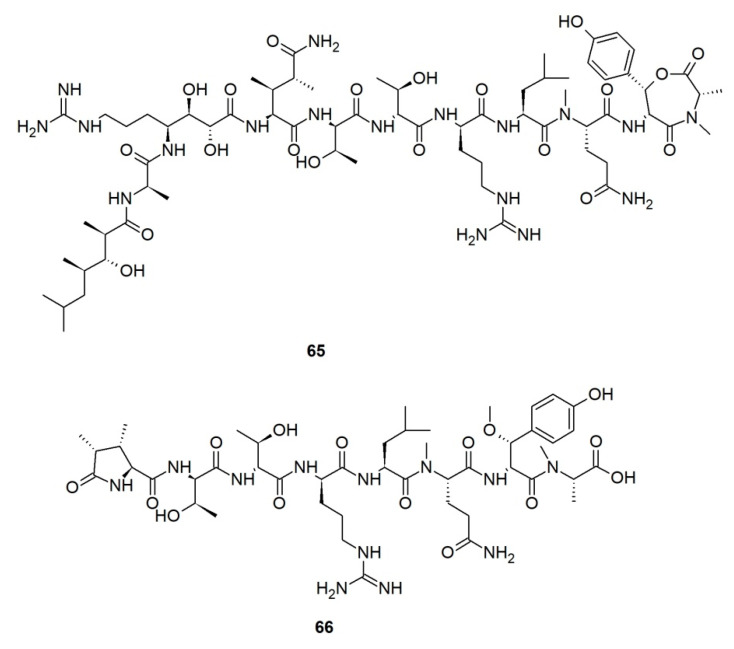
Callipeltins reported from the genus *Latrunculia*.

Callipeltins are a small family of marine peptides comprising 17 members (callipeltins A–Q), 13 of which (callipeltins A–M) originate from an unidentified *Latrunculia* sp., collected in Vanuatu, Oceania (Figure 8) [42,43,44,101]. Structurally, callipeltins are characterized by the presence of several non-proteinogenic units. For instance, the cyclic decapeptide callipeltin A (**54**) contains three unusual amino acid residues, β-methoxytyrosine (β-OMeTyr), (2*R*,3*R*,4*S*)-4-amino-7-guanidino-2,3-dihydroxyheptanoic acid (AGDHE), and (3*S*,4*R*)-3,4-dimethyl-l-glutamine (diMeGln) [42]. Some other unusual amino acids are represented by the 3,4-dimethyl-l-pyroglutamic acid (the *N*-terminus unit) in callipeltin B (**55**); d-arginine in callipeltin F (**59**); l-*N*-methylglutamine in callipeltins J (**63**) and K (**64**) [44]. 

Callipeltin A (**54**) was originally isolated from a New Caledonian *Callipelta* sp. (now accepted as an unidentified specimen of *Sollasipelta* Van Soest & Hooper, 2020 [102] (family Neopeltidae Sollas, 1888 [103], order Tetractinellida Marshall, 1876 [104]). D’Auria group reported callipeltins A–C (**54**–**56**) together with two new acyclic peptides, callipeltins D (**57**) and E (**58**) (Figure 8), from a *Latrunculia* sp. from Vanuatu [42]. Marfey’s reagent was used to establish the absolute stereochemistry of the new compounds and for the structural revision of callipeltin A (**54**). Callipeltin A (**54**) and B (**55**) appeared to exert strong cytotoxicity against multiple cell lines, including human bronchopulmonary non-small cell lung carcinoma (NSCLC-N6); human renal carcinoma (E39), murine leukemia (P388), and human melanoma (M96), by showing IC_50_ values ranging from 1.1 to 10 μg/mL, while callipeltin C (**56**) was only moderately cytotoxic against NSCLC-N6 and E39 cells (IC_50_ values 53.5 and 36.1 μg/mL, respectively). Callipeltins A (**54**) and B (**55**) exhibited in vitro antifungal activity against *C. albicans* on a disc diffusion assay. Callipeltin A (**54**) also showed in vitro anti-HIV activity (SI = 29) [105,106] and is a selective and significant inhibitor of the Na^+^/Ca^2+^ exchanger and a positive inotropic agent [107]. Further investigations on the same sponge yielded callipeltins F–M, (**59**–**66**) [43,44]. Callipeltins F–K (**59**–**64**) were found to display antifungal activity towards *C. albicans* (MIC values of ca. 10^−4^ M) but lacked anti-HIV activity [43,44]. Until now, this is the only *Latrunculia* sp. that has been reported to contain these unique marine peptides. 

#### 3.1.6. Other Types of Compounds 

A few other classes of MNPs were reported from sponges that were originally loosely grouped in the genus *Latrunculia*. Between 1985 and 1998, the Capon group studied several Australian sponges initially identified as *Latrunculia* sp. and reported a suite of norditerpene or norsesterterpene cyclic peroxides with the trivial names of trunculins and conulosins [46,47,108,109,110,111]. Further taxonomic revisions have reidentified the sponge specimens as species of *Sigmosceptrella* Dendy, 1922 (family Podospongiidae de Laubenfels, 1936) [49,50,83]. He et al. (1991) also reported three new trunculins in their methyl ester form from an Australian specimen of *Latrunculia* [112]. Based on its sampling site (Jervis Bay, Australia) and chemical composition, this sponge could potentially be a specimen of either *Diacarnus* Burton, 1934 [113] or *Sigmosceptrella*. Cheenpracha et al. (2010) investigated an Indo-West Pacific specimen identified as *Latrunculia* sp., which yielded seven norsesterterpene peroxides (epimuqubilin A, muqubilone B, unnamed cyclic peroxide ester, epimuqubilin B, sigmosceptrellin A methyl ester, sigmosceptrellin A, sigmosceptrellin B methyl ester, and an unnamed cyclic peroxide ester) [114]. Although no taxonomic investigation has been carried out, this sponge appears to be misidentified based on the chemical composition. Kelly-Borges and Vacelet (1995) described seven species of *Diacarnus* from the Indo-West and Central Pacific [115], and a species of this genus (or *Sigmosceptrella*) most likely to be the correct identity. Currently, trunculins or conulosins are regarded as biochemical markers important for chemotaxonomy of the sponge genera *Diacarnus* and *Sigmosceptrella* [50].

Latrunculins are a group of marine toxins belonging to the polyketide macrolide family previously reported from the Red Sea sponge *Latrunculia magnifica* Keller, 1889 [116], which is now accepted as the podospongid *Negombata magnifica* Keller, 1889 [45,117,118,119].

### 3.2. Genus Strongylodesma 

With nine reported species so far, *Strongylodesma* represents the second-largest genus of the family Latrunculiidae. Although they lack the diagnostic anisodiscorhabds that differentiate species of other latrunculid genera, the possession of smooth or terminally spined strongyle megascleres, in a single, broad size category, many of which have characteristic “shepherd’s crook” modifications, serve to distinguish *Strongylodesma* species from each other and from other latrunculid sponge genera and their species [57]. The strongyles form an irregular, large-meshed reticulation of heavy or loose tracts, that are somewhat reminiscent of the honeycomb structures in species of *Tsitsikamma*. The description of the first species from Australian waters, *Strongylodesma australiense* Kelly & Goudie, 2020 [59], from New South Wales, is significant, because it is the first record of *Strongylodesma* in the Southwest Pacific, providing a bridge between the disparate tropical Western and south Atlantic Ocean, and the western Pacific Ocean locations of other species (Kelly & Goudie 2020) [59]. *Strongylodesma* species are found both in shallow water (e.g., intertidal pools) and the deep-sea (exceeding −100 m) [25,57,120] and their morphology and coloration is very similar to that of other Latrunculiidae [57]. The genus *Strongylodesma* is another very prolific source of discorhabdin-type alkaloids and all known *Strongylodesma* species produce pyrroloiminoquinone (discorhabdins) and their structurally related alkaloids. 

Between 1987 and 1991, Kobayashi group reported four sulfur-containing pyrroloiminoquinone alkaloids, prianosins A–D (**2**, **67**, **7**, and **4**, Figure 3) from an Okinawan sponge identified as *Prianos melanos* de Laubenfels, 1954 [91,121,122] on account of the possession of strongyles with shepherd’s crook modifications. However, this sponge was revised as *Strongylodesma melanos* by Samaai et al. in 2009 [57,91,121,122]. The structure of (+)-prianosin A was unambiguously determined by X-ray diffraction analysis [91]. The report of discorhabdins with the same NMR data from New Zealand *Latrunculia* sp. around the same period with prianosins [69,71] pointed out that (+)-prianosin A/(+)-discorhabdin A (**2**), (+)-prianosin C/(+)-2-hydroxydiscorhabdin D (**7**), and (+)-prianosin D/(−)_546_-discorhabdin D (**4**) are chemically identical compounds [91,121,122]. (+)-Prianosin B (16,17-dehydrodiscorhabdin A, **67**), however, has never been reported from any other latrunculid sponge. Prianosins A–D show strong in vitro anticancer potency against mouse lymphocytic leukemia (L1210) and murine leukemic lymphoblast (L5178Y) cell lines with IC_50_ values of 0.037 and 0.014 μg/mL (prianosin A, **2**), 2.0 and 1.8 μg/mL (prianosin B, **67**), 0.15 and 0.024 μg/mL (prianosin C, **7**) and 0.18 and 0.048 μg/mL (prianosin D, **4**) [91,121,122]. Prianosins C (**7**) and D (**4**) were also cytotoxic against human epidermoid carcinoma (KB) cells (IC_50_s 0.57 and 0.46 μg/mL, respectively) while prianosins A (**2**) and D (**4**) significantly induced Ca^2+^ release from the sarcoplasmic reticulum [91,121,122].

Two Caribbean species, identified as species of *Batzella* (−141 m depth) from which new discorhabdins P (**68**), S–U (**69**–**71**) (Figure 3) were obtained [85,123] were revised as *S. purpurea* in 2009 [57]. Discorhabdin P (**68**) is an N-13 methylated derivative of discorhabdin C, while discorhabdins S–U (**69**–**71**) are chiral molecules with one stereocenter at C-6 and bear an additional S-methyl group at C-5 (Figure 3) [123]. This study did not report the [α]_D_ values of the compounds. The absolute configurations of compounds **69**–**71** were only determined in 2010 as 6*S* via semisynthesis [75]. Discorhabdins P, S–U (**68**–**71**) were evaluated for their in vitro cytotoxicity against various cell lines, i.e., murine P-388, human lung carcinoma (A-549) cell line and human pancreatic cancer cell (PANC-1). As shown in Table 2, discorhabdins P (**68**) and U (**71**) are the most potent [123]. Discorhabdin P (**68**) is an inhibitor of calcineurin (CaN) and caspase-3 (CPP32) enzymes (IC_50_ values 1.15 and 0.77 μM), suggesting the great potency of discorhabdin P in preventing the pathological damage induced by caspase-mediated apoptotic events [85]. 

The Davies-Coleman group reported known discorhabdin alkaloids (+)-discorhabdin A (**2**), (−)_546_-discorhabdin D (**4**), 3-dihydrodiscorhabdin C (**26**), and (−)-discorhabdin H (**9**) as major components of the South African sponge *S. algoaensis* Samaai & Kelly, 2003 (Figure 3) [30]. This was the first report of the fully assigned 1D NMR data of discorhabdin H (**9**). Compound **9** bears the typical heptacyclic discorhabdin d-skeleton substituted with an unusual histidine moiety at C-1 and the absolute configuration of the histidine moiety was assigned by chiral GC-MS analysis of the acylated ozonolysis products of discorhabdin H (**9**) [30]. When tested against human colon tumor cell line HCT-116, compound **2** was found to be the most potent molecule (IC_50_ value of 7.0 nM). 

#### 3.2.1. Batzellines and Isobatzellines

Sakemi et al. (1989) reported batzellines A (**72**), B (**73**), and C (**52**) from a deep-sea Caribbean sponge *Batzella* sp. collected at a depth of 120 m (Figure 7) [124]. A second collection of *Batzella* sp. (1990) from the same region yielded isobatzellines A–D (**74**–**77**) (Figure 7) [125]. In 2009, both sponge specimens were reidentified as *S. nigra* Samaai & Kelly, 2009 by Samaai et al. [57]. The structure of batzelline A (**72**) was ascertained by X-ray analysis. Isobatzellines have very similar backbone to batzellines with a further amination at C-7, hence, forming the characteristic pyrrolo[4,3,2-*de*]quinoline ring system for discorhabdins [68,125]. All batzellines and isobatzellines, except for isobatzelline B (**75**) reported in this study are chlorinated compounds. Interestingly, all batzellines and isobatzellines bear an S-methyl on C-2 and/or an *N*-methyl on N-1, which are uncommon for (bis)pyrroloiminoquinones and their analogs [125]. Isobatzellines (**74**–**77**) but not batzellines A–C (**72**, **73**, and **52**), were found to exert cytotoxic and antifungal activities [124,125], however, their IC_50_ or MIC values were not reported.

#### 3.2.2. Makaluvamines, Damirones, Makaluvic Acids, and Tsitsikammamines

Polar extracts of a new South African sponge, *S. aliwaliensis* Samaai, 2004 [120] yielded the known alkaloids damirone C (**78**), makaluvamines I (**79**) and M (**80**), and four new compounds: makaluvic acid C (**81**), *N*-1-β-d-ribofuranosyldamirone C (**82**), *N*-1-β-d-ribofuranosylmakaluvamine I (**83**), and *N*-1-β-d-ribofuranosylmakaluvic acid C (**84**) (Figure 7) [126,127]. The sugar residues were confirmed by GC-MS analysis of the peracetylated aldononitrile derivatives of free sugars obtained by hydrolysis of compounds **82**–**84** and comparison with that of four aldopentoses pretreated with the same derivatization. Compounds **82**–**84** are also the first and the only glycosylated alkaloids reported from latrunculid sponges so far. Compounds **80** and **82** displayed sub-micromolar activity against the esophageal cancer cell line WHCO-1 (IC_50_ values 0.7 and 1.6 μM, respectively), whereas **78**, **81**, and **84** were marginally active (IC_50_ values 56, 38, 61 μM, respectively) [126,127].

An investigation on *S. tongaensis* Samaai & Kelly, 2009 [57] afforded the new alkaloids 6-bromodamirone B (**85**) and makaluvamine W (**86**), plus eight known compounds, makaluvamines A (**87**), E (**88**), F (**53**), and K (**89**), makaluvone (**90**), damirone B (**91**), makaluvic acid A (**92**) and tsitsikammamine B (**93**) (Figure 5 and Figure 7) [128]. Similar to citharoxazole (**51**) [66], makaluvamine W (**86**) bears an oxazole function on the aromatic ring. Compound **86** represents the first makaluvamine-type alkaloid with a nitrogen substitution at C-8. All compounds were tested against the human promyelocytic leukemia cell line HL-60. Compounds **53**, **89**, **90**, and **93** were the most active, with IC_50_ values of 3.0, 4.5, 2.6, and 1.6 μM, respectively, while **87** and **89** had much lower activity (IC_50_ values 30.4 and 20.3 μM, respectively) [128]. 

### 3.3. Genus Tsitsikamma

*Tsitsikamma* is a small genus of latrunculid sponges, endemic to South Africa, the name *Tsitsikamma* having been derived from the type locality, the marine protected area (MPA) of The Garden Route National Park named Tsitsikamma [129]. The genus currently comprises eight species, with the addition of three new species in two new subgenera (*Tsitsikamma* Samaai and Kelly, 2002 and *Clavicaulis* Samaai, Payne and Ngwakum, 2020) [41]. Members of the genus *Tsitsikamma* are mainly found in shallow water down to 25 m depth [25]. The genus *Tsitsikamma* has been regarded as a morphological intermediate between *Latrunculia* and *Zyzzya* [25]. Species of *Tsitsikamma* exhibit a similar morphology to other latrunculid sponges, especially the aquiferous structures, but they have a tough and leathery texture due to the sponge’s huge internal spicule tracts, which promote a purse- or honeycomb-like architecture [25]. Sponges of this genus are prolific sources of discorhabdins and makaluvamines.

#### 3.3.1. Discorhabdins 

*Tsitsikamma* (*Tsitsikamma*) *favus* Samaai & Kelly, 2002 [25] yielded the first examples of discorhabdins with a C-14 substituent, i.e., 14-bromodiscorhabdin C (**94**) and 14-bromo-3-dihydrodiscorhabdin C (**95**) (Figure 3) [99]. The configuration of the stereogenic center at C-3 remains unknown and its [α]_D_ data were not recorded. Both compounds **94** and **95** were reported to exhibit antimicrobial activity against *B. subtilis* without reported MIC or IC_50_ values [99].

The examination of the organic extracts of two South African *Tsitsikamma* species, *Tsitsikamma* (*Clavicaulis*) *pedunculata* Samaai & Kelly 2003 [56] and *T*. (*T*.) *favus* Samaai & Kelly, 2002, yielded three known and four new discorhabdin C-and discorhabdin V-type alkaloids [30]. Known discorhabdin C analogs **94**, **95,** and **26** and the new analogs 3-dihydro-7,8-dehydrodiscorhabdin C (**32**), 14-bromo-3-dihydro-7,8-dehydrodiscorhabdin C (**96**), discorhabdin V (**97**), and 14-bromo-1-hydroxydiscorhabdin V (**98**) were sourced from *T.* (*C*.) *pedunculata* Samaai & Kelly 2003 (Figure 3). The *T*. (*T*.) *favus* Samaai & Kelly 2003 specimen produced the known compound **95** and the new metabolites **32** and **98** (Figure 3) [30]. The new discorhabdins contain one or more stereocenter(s), however, their configuration was not assigned. When tested against the human colon cancer cell line HCT-116, 14-bromodiscorhabdin C (**94**) appeared as the most potent molecule (IC_50_ value 77 nM), while 14-bromo-1-hydroxydiscorhabdin V (**98**) was the weakest (IC_50_ value 12.5 μM). 

Kalinski et al. reported the reisolation of 14-bromo-3-dihydro-7,8-dehydrodiscorhabdin C (**96**) from *T*. (*T*.) *favus* Samaai & Kelly, 2002 [40]. The authors also demonstrated topoisomerase I inhibition, DNA intercalation and moderate in vitro anticancer and cytotoxic potential of **96** against human embryonic kidney 293 (HEK 293) and HeLa cells [40].

#### 3.3.2. Tsitsikammamines, Makaluvamines and Makaluvone 

The first members of the tsitsikammamine family, tsitsikammamines A (**47**) and B (**93**) were reported from a South African sponge *T*. (*T*.) *favus* Samaai & Kelly, 2002 collected from the Tsitsikamma Marine Reserve [99]. In vitro bioactivity tests revealed antimicrobial activity of these two compounds against *B. subtilis*. Tsitsikammamines were considered as strong chemotaxonomic markers for *Tsitsikamma* species until the report of tsitsikammamine C (**101**) from an Australian *Zyzzya* sp. and the absence of tsitsikammamines in the South African sponge, *T.* (*C*.) *pedunculata* Samaai & Kelly 2003 [30,100]. As chemotaxonomic markers for the genus *Tsitsikamma*, a critical role was placed to 14-brominated discorhabdin analogs which are present in all studied *Tsitsikamma* spp. so far [30,49]. Antunes et al. examined the chemical profile of two South African *Tsitsikamma* species. Tsitsikammamines A (**47**) and B (**93**), and their respective *N*-oxime analogs **99** and **100**, were obtained from *T*. (*T*.) *favus* Samaai & Kelly, 2002 while *T.* (*C*.) *pedunculata* Samaai & Kelly 2003 only contained discorhabdin-type alkaloids (Figure 5, See Section 3.3.1) [30]. The *N*-oxime analogs **99** and **100** were deemed as isolation artifacts of the naturally occurring tsitsikammamines A (**47**) and B (**93**) *N*-oxides [30]. Both tsitsikammamines A (**47**), B (**93**) and their *N*-oxime analogs **99** and **100** exhibited similar topoisomerase I inhibitory activity and intercalated DNA with *K*s values (the micromolar concentration that decreases DNA-bound ethidium bromide fluorescence by 50%) of 15, 45, 20, and 30 μM, respectively [30]. Against HCT-116 cells, tsitsikammamines A (**47**) and B (**93**) exerted promising cytotoxicity with IC_50_ values of 1.4 and 2.4 μM, respectively, while the *N*-oximes **99** and **100** were weaker (IC_50_s 128.2 and 16.5 μM, respectively). 

In 2019, Kalinski et al. studied the secondary metabolite profile of seven South African *T.* (*T*.) *favus* Samaai & Kelly, 2002 specimens [40]. HR-ESI-LC-MS/MS-based MN studies revealed the presence of two chemotypes; chemical composition of chemotype I was predominated by discorhabdins and tsitsikammamines, while specimens of chemotype II produced mainly halogenated makaluvamines and trace levels of tsitsikammamines [40]. Chemical work-up of a chemotype I sponge *T.* (*T*.) *favus* Samaai & Kelly, 2002 sponge yielded tsitsikammamine B (**93**) and a chemotype II *T.* (*T*.) *favus* Samaai & Kelly, 2002 yielded the new compound makaluvamine Q (**102**) together with four known metabolites, namely, makaluvamines A (**87**), I (**79**), and O (**103**) and makaluvone (**90**) (Figure 7) [40]. Makaluvamine Q **(102**) represents the first and the only example of an *N*-methylated makaluvamine with a bromination on C-6. This study reported the presence of makaluvamines in the genus *Tsitsikamma* for the first time. All seven compounds showed some topoisomerase I inhibitory and DNA intercalation activities. Makaluvamine O (**103**) and makaluvone (**90**) appeared as the most effective topoisomerase I inhibitors and the weakest DNA intercalators [40], and they exerted the weakest activity against human embryonic kidney 293 (HEK 293) and HeLa cells. Makaluvamine Q (**102**) exhibited the highest DNA affinity and also showed the strongest in vitro activity against HEK 293 and HeLa cells and their IC_50_ values were not recorded [40]. This study suggested that DNA intercalation, instead of topoisomerase I, might be the anticancer targets of the planar molecules makaluvamines (**79**, **87**, **102**, and **103**) and makaluvone (**90**) [40]. 

### 3.4. Genus Sceptrella 

The genus *Sceptrella* is restricted to three species: the type species, *S*. *regalis* Schmidt, 1870 [23]; *S*. *biannulata* Topsent, 1892 [130] and *S*. *insignis* Topsent, 1890 [131], all from the Atlantic Ocean, specifically Floridian waters, and the Azores archipelago [25,132]. *Sceptrella* was compared to the monospecific Aleutian Islands genus, *Latrunculava* Kelly, Reiswig & Samaai, 2016, also with a second spicule form, the “sceptre”, in addition to the anisodiscorhabd first recorded from the Oamaru Diatomite (Eocene) of New Zealand [21]. Species of *Sceptrella* are morphologically similar to *Latrunculia* and *Strongylodesma* species, with a cakey, dense and compressible texture, and a hemispherical or spherical body shape [132]. *Sceptrella* species are deep-sea organisms, having been recorded from a depth of 2460 m in some regions (e.g., the north coast of Norway) [25]. Biochemical studies on sponges of the genus *Sceptrella* yielded only pyrroloiminoquinones (discorhabdins) and structurally related alkaloids (makaluvamines). To date, there has only been one chemical study from a sponge purported to be a species of *Sceptrella*, from Korea (potentially a species of *Latrunclava*), that yielded two new discorhabdin alkaloids, (−)-3-dihydrodiscorhabdin D (**104**) and (−)-discorhabdin Z (**105**) together with 12 previously reported pyrroloiminoquinones and related alkaloids namely, discorhabdin C (**1**), didebromodiscorhabdin C (**106**), 3-dihydrodiscorhabdin C (**26**), (+)-discorhabdin E (**5**), (+)-discorhabdin B (**3**), (+)-discorhabdin G*/I (**25**), (−)_546_-discorhabdin D (**4**), (+)-1-methoxydiscorhabdin D (**107**), (−)_546_-discorhabdin L (**13**), (−)-dihydrodiscorhabdin L (**108**), makaluvamine D (**109**) and (−)-makaluvamine F (**53**) (Figure 3 and Figure 7) [133]. The 2D structures of the new compounds were established by conventional spectroscopic methods, while the absolute configuration was then assigned by their [α]_D_ values and ECD spectra in conjunction with DFT-calculations [133]. Most of the isolated metabolites exhibited cytotoxicity and antimicrobial activity. The strongest antibacterial activity was shown by (+)-discorhabdin B (**3**) against *Proteus vulgaris* (MIC 3.13 μg/mL) and the strongest cytotoxicity was exerted by (+)-discorhabdin E (**5**) (IC_50_ values 1.3 μM) [133]. (−)-Discorhabdin Z (**105**) inhibited the sortase A enzyme, an antibacterial drug target, with an IC_50_ value (6.5 μM) that was almost 20 times lower than the positive control *p*-(hydroxymercury)benzoic acid (IC_50_ 110.9 μM) [133]. 

### 3.5. Genus Cyclacanthia

The genus *Cyclacanthia* is also endemic to South African waters and is represented by four species: *Cyclacanthia mzimayiensis* Samaai & Kelly, 2004 and *C. cloverlyae* Samaai & Kelly, 2004 [26] were reported from the subtropical east coast of South Africa, and the type species *C. bellae* Samaai & Kelly, 2003 was described from the temperate Algoa Bay [26,56]. A fourth species, *C*. *rethahofmeyri* Samaai, Payne and Ngwakum & Kelly is reported from South Africa [41]. To date, there has been only one report on the chemical constituents of the genus *Cyclacanthia*. New discorhabdins 1-methoxydiscorhabdin D (**107**) and 1-aminodiscorhabdin D (**110**) together with five known metabolites discorhabdins N (**15**) and I (**25**), damirone B (**91**), makaluvic acid A (**92**), and makaluvamine C (**111**) were obtained (Figure 3 and Figure 7) [30] from *Latrunculia bellae* Samaai & Kelly, 2003 (now accepted as *Cyclacanthia bellae* Samaai & Kelly, 2003) [26]. A re-examination of this sponge extract by Grkovic et al. in 2008 yielded another known compound (−)_546_-discorhabdin L (**13**) [74]. Compounds **15, 107,** and **110** bear the same heptacyclic skeleton as discorhabdin D (**4**) but vary from each other with different substitutions at C-1. The relative configuration of C-1, absolute configurations and optical rotations of the molecules were not reported. Compounds **107**, **110**, **25** were clearly the most cytotoxic against human colon tumor cell line (HCT-116) with IC_50_ values 0.23, 0.12, and 0.33 μM, respectively. Compounds **15**, **91**, and **111** exhibited moderate cytotoxicity against HCT-116 cells with IC_50_ values ranging from 1.1 to 3.1 μM, while **92** had modest activity (IC_50_ value 28.4 μM) [30] 

### 3.6. Other Genera

Apart from the five genera *Latrunculia*, *Strongylodesma*, *Tsitsikamma*, *Sceptrella*, and *Cyclacanthia*, the family Latrunculiidae includes several new genera established in 2016: *Bomba* Kelly, Reiswig & Samaai, 2016 and *Latrunclava* Kelly, Reiswig & Samaai, 2016 [21]. Genus *Bomba* comprises two species: the type species *B. tricincta* Hentschel, 1929 (initially identified as *Latrunculia tricincta* Hentschel, 1929) [134] reported from northern Norway, and the species *B. endeavourensis* Kelly, Reiswig & Samaai, 2016 [21] discovered in deep waters on Endeavour Ridge, British Columbia, Canada. Genus *Latrunclava*, discussed above in a comparison with genus *Sceptrella,* was established with only one species, *Latrunclava imago* Kelly, Reiswig & Samaai, 2016 [21], which was collected from Amchitka Pass, in the central Aleutian Islands. So far, no chemical investigation has been carried out on any of the new latrunculid sponge genera *Bomba* and *Latrunclava* because they are monospecific and restricted to small holotype species. 

## 4. Biosynthetic Origin of Latrunculid Sponge Metabolites

Marine sponges are holobionts, comprising the sponge host cells and a dense microbiome. Sponge-associated microorganisms account for a large proportion of the sponge biomass, varying from 30% to 60% in total [135,136,137]. Significant physiological and ecological roles have been ascribed to sponge-associated microorganisms, including nutritional enhancements, stabilization of the sponge skeleton, and most importantly, production of secondary metabolites [138,139,140]. Hence, the true natural origin of sponge-derived metabolites is one of the key areas of sponge natural product research. 

The debate on the origin of latrunculid sponge-derived pyrroloiminoquinone and bispyrroloiminoquinone alkaloids started shortly after the first report of discorhabdin C from an unidentified New Zealand species of *Latrunculia* [27], and still continues [40]. In 1991, Copp et al. reported the isolation of a new alkaloid wakayin from the colonial ascidian *Clavelina* sp. [36]. Wakayin belongs to the bispyrroloiminoquinone family and bears the same pyrrolo[4,3,2-*de*]quinoline ring system as latrunculid sponge metabolites, isobatzellines A–D, makaluvamines and discorhabdins [27,69,71,125]. Biosynthetically, isobatzellines, discorhabdins, and wakayin were proposed to be derived from the same precursor tryptophan [34,40]. The report of wakayin from a phylogenetically distinct ascidian indicated that bis-pyrroloiminoquinone-type alkaloids are not confined to the phylum Porifera. The report of wakayin also raised the question as to whether bispyrroloiminoquinone-type alkaloids originated from the sponge hosts or the associated microbes [36]. 

**Scheme 1:**
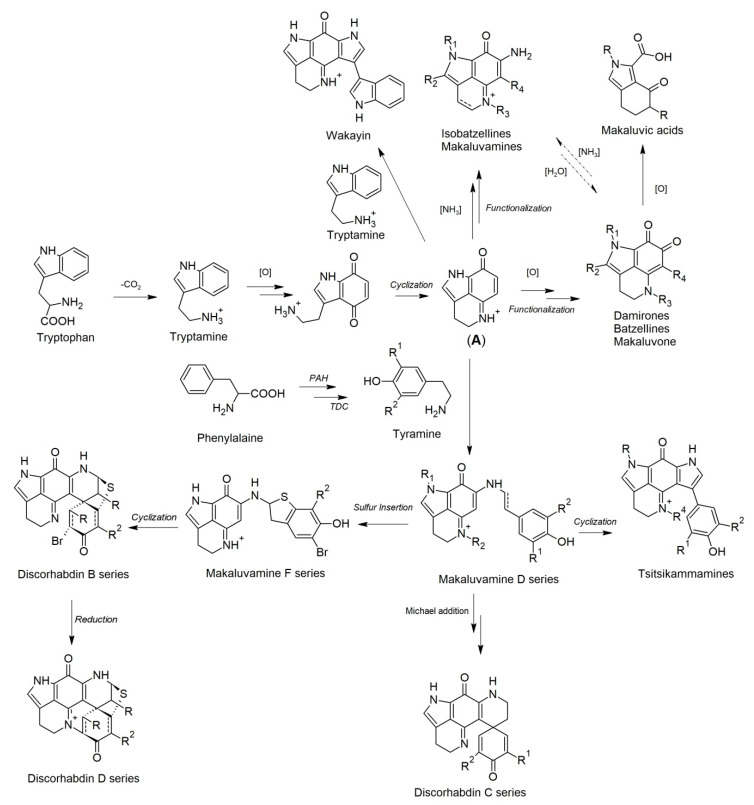
Proposed biosynthetic pathway of discorhabdins and structurally related metabolites (adapted from references [34,40,49,82,141]).

In 1995, Lill et al. carried out a preliminary investigation on the production of discorhabdin B using slices and unfractionated dispersed cells of the sponge *Latrunculia* sp. [34]. A putative biosynthetic pathway was proposed based on the perceived chemotaxonomic relationships between various groups of (bis)pyrroloiminoquinones found in different marine organisms. In this biogenesis, decarboxylation of tryptophan formed tryptamine, from which the pyrroloquinoline backbone (**A**) was generated after appropriate functionalization and oxidation (Scheme 1). The parent pyrroloquinoline (**A**) is considered to be the direct precursor of simple discorhabdin-related alkaloids, e.g., damirones, batzellines, and isobatzellines (Scheme 1). Via the incorporation of tyramine or a functionalized tyramine derivative, the parent pyrroloquinoline (**A**) then led directly to more complex pyrroloiminoquinone and their analogs, including discorhabdins and makaluvamine D series alkaloids (Scheme 1). To verify the proposed biosynthetic pathway, thin slices of a discorhabdin B-producing *Latrunculia* specimen were incubated with isotopically labeled phenylalanine ([U-^14^C]-l-phenylalanine). The de novo synthesis of discorhabdin B utilizing [U-^14^C]-l-phenylalanine was clearly observed by the detection of ^14^C via radiochemical analysis in the purified discorhabdin B, hence further confirming the biosynthetic pathway proposed by Lill et al. Aubart et al. (1999) advanced the putative biosynthetic pathway suggested by Lill et al. based on their successful syntheses of discorhabdins C and E and dethiadiscorhabdin D via a biomimetic approach [141]. In this advanced biosynthesis, the discorhabdin C skeleton was formed by an intramolecular Michael addition of makaluvamine D followed by auto-oxidation back to the quinone, and the discorhabdin D skeleton was formed by nucleophilic displacement of a C2 bromide to generate the C2–N18 bond, following the 1,5-hydride addition of discorhabdin B (Scheme 1) [141]. The wakayin skeleton was formed by a further substitution of tryptamine on the pyrroloquinoline backbone (**A**) and the tsitsikammamine skeleton was formed by an alternative cyclization of makaluvamine D (Scheme 1) [82]. Batzellines and isobatzellines are convertible via amination and hydrolyzation (Scheme 1) [40]. Through the modified biogenetic proposal raised by Lill, Aubart, Urban, and Kalinski, the whole suite of known monomeric discorhabdins and structurally related metabolites could be generated (Scheme 1).

In order to address the question of whether the biosynthesis of pyrroloiminoquinones is restricted to the sponge cells or sponge-associated microbes alone, or requires the cooperation of commensal microorganism in its production, Lill et al. (1995) cultured slices of discorhabdin B-producing sponge (*Latrunculia* sp.) tissue with [U-^14^C]-l-phenylalanine under three different conditions: (**A**) the sponge tissue slices were presoaked with a broad-spectrum antibiotic mixture and then incubated in an artificial medium; (**B**) presoaked sponge tissue slices were incubated with broad-spectrum antibiotics; and (**C**) sponge tissue slices were not subjected to any antibiotic treatment [34]. The results revealed that the ^14^C-incorporation into discorhabdin B in both groups **A** and **B** was similar to that in group **C**, indicating that the production of discorhabdin B does not require the presence of sponge-associated bacteria [34]. This is the first and the only in vivo study referring to the natural origin of discorhabdins. Interestingly, in 1996, Hooper et al. reported the isolation of two bispyrroloiminoquinones (tsitsikammamines A and B) from a South African latrunculid sponge (*Tsitsikamma* sp.), while Ishibasha et al. (2001) reported a structurally related compound makaluvamine A from a laboratory cultured myxomycete *Didymium bahiense* [38,99]. Both tsitsikammamines and makaluvamine A bear the identical pyrrolo[4,3,2-*de*]quinoline ring system as wakayin does. The wide distribution of pyrroloiminoquinone and their structural related alkaloids in taxonomically unrelated organisms such as Porifera, tunicate, and myxomycete, suggest a possible symbiont origin for this type of compounds (especially to tsitsikammamines) although the production of discorhabdin B in sponge cells has been proved by Lill and coworkers [34]. 

A culture-independent study by Walmsley et al. investigated the diversity of bacterial communities associated with three *T*. (*T*.) *favus* Samaai & Kelly, 2002 specimens from South Africa [142]. An NMR-based metabolomics study revealed the major compound to be tsitsikammamine B for all three samples. The dominating bacterial species of these three samples was identified to be a unique Betaproteobacterium using denaturing gradient gel electrophoresis (DGGE) together with clonal and deep amplicon sequencing of the microbial 16S rRNA gene. This is the first report of β-proteobacteria as the dominant taxon in a sponge holobiont, however, whether this β-proteobacterial community is responsible for the production of tsitsikammamines remains undetermined [142]. A subsequent study by Matcher et al. (2016) investigated whether the β-proteobacterium operational taxonomic unit (OTU_0.03_) dominates the bacterial community in other sponge species which produce (bis)pyrroloiminoquinones [143]. Hence, the microbial composition of nine sponges represented by eight latrunculid sponge specimens (three *T*. (*T*.) *favus* Samaai & Kelly, 2002 specimens, three unidentified *Tsitsikamma* specimens*,* one *L.* (*B.*) *algoaensis* Samaai, Janson & Kelly, 2012, and one *Cyclacanthia bellae* Samaai & Kelly, 2003) and one *Mycale* sp. specimen (found encrusting on one of the *Tsitsikamma* specimens) was investigated [143]. They observed the conservation of a single, dominant β-proteobacterium OTU within the microbiome of all eight latrunculid sponges (also at low levels in the seawater but not in the sediment) [143]. The microbial community of the *Mycale* sp. was dominated by a different β-proteobacterium species. The phylogeny of all these β-proteobacterial OTUs closely resembles that of their host sponges, suggesting that these Gram-negative bacteria may have coevolved with their hosts. The β-proteobacterial OTUs are numerically dominant and metabolically active in both *T*. (*T*.) *favus* Samaai & Kelly, 2002 and *C. bellae* Samaai & Kelly, 2003. Sequence analysis (BLAST) of the 16S rRNA genes from these β-proteobacterial strains identified them to be Nitrosomonadaceae bacteria, which have been proven to be lithoautotrophic ammonia oxidizers [144]. Furthermore, a significant proportion of Spirochaetae bacteria are also present in all nine sponges except for the *Mycale* sp. and *L.* (*B.*) *algoaensis* Samaai, Janson & Kelly, 2012 specimens [143]. The Spirochaetae bacteria found in sponges of the genera *Tsitsikamma* and *Cyclacanthia* were dominated by a single OTU_(0.03)_ that have a close phylogenetic relationship with each other and a distant relationship to Spirochaetae bacteria found in other sponge families. Hence, the authors speculated the involvement of these Spirochaetae OTUs in the pyrroloiminoquinone production of sponges of the genera *Tsitsikamma* and *Cyclacanthia* [143]. 

Although the latrunculid sponge genera *Tsitsikamma*, *Cyclacanthia*, and *Latrunculia* share a similar bacterial community, tsitsikammamines have never been reported from a sponge of the genus *Latrunculia* until our report in 2018. Therein, a comprehensive MN-based metabolomic study of an Antarctic *Latrunculia* sp. revealed tsitsikammamines as the major component of the sponge [67]. The presence of tsitsikammamines in a species of *Latrunculia* lends support for the potential microbial origin of tsitsikammamines and contributes to discussions on the biosynthetic origin of (bis)pyrroloiminoquinones. A microbial community profiling of ten South African sponges of the genus *Tsitsikamma* revealed the conservation of a dominant, single β-proteobacterium OTU_0.03_ and a second dominant Spirochetes OTU_0.03_ within all the sponge specimens [40]. The authors hypothesized that the production of (bis)pyrroloiminoquinones was a combined effort of the sponge host and its selected associated microbes; after the production of makaluvamines and parent pyrroloquinoline backbone by the microbes (**A**, Scheme 1), the sponge hosts convert these simple precursors to more complex metabolites, i.e., discorhabdins and tsitsikammamines [56]. The debate on the biosynthetic origin of (bis)pyrroloiminoquinones is still ongoing, and it is tenuous to make a definitive conclusion. 

## 5. The Significance of Pyrroloiminoquinone-Type Alkaloids in the Chemotaxonomy and Phylogeny of Latrunculid Sponges

The distribution of norsesterterpene cyclic peroxides and pyrroloiminoquinone alkaloids across genera previously considered to be of latrunculid origin facilitated the separation of norsesterterpene cyclic peroxide-containing genera *Sigmosceptrella*, *Diacarnus* and *Negombata* Laubenfels, 1936 [51], from pyrroloiminoquinone-containing genera *Latrunculia*, *Sceptrella* and related taxa, into separate families within the order Poecilosclerida. This scheme was initially hypothesized by Kelly in Urban et al. (2000) [82], and formalized by the resurrection of the family Podospongiidae by Kelly and Samaai (2002) [145], to receive norsesterterpene cyclic peroxide-containing genera, resulting in a monophyletic group well separated from family Latrunculiidae.

The process undertaken provides a good example of the power of marine natural products to strengthen our understanding of the phylogeny of specific sponge groups. The thorough taxonomic investigation and full identification and description of all host and source sponges, by experts, underpins and facilitates such research. The process is of inestimable value to marine natural product studies; taxonomy underpins and smooths the biodiscovery process, allowing accurate drug discovery predictions and dereplication of samples, saving time and precious funding.

The need for good taxonomy is illustrated in the discovery that shepherd’s crook modifications of strongyles are diagnostic for latrunculid *Strongylodesma* and *Batzella* Topsent, 1893 (order Poecilosclerida, family Chondropsidae) only (see Kelly and Goudie, 2020) [59], but not for species of *Prianos* Gray, 1867 [146] (order Haplosclerida Topsent, 1928, family Chalinidae). *Prianos* (now accepted as *Haliclona* (*Reniera*) Schmidt, 1862) [147] is a genus to which many latrunculid species had been mistakenly assigned, resulting in serious confusion of species in the literature and confusion in the nomenclature of discorhabdins.

The full taxonomic process is exemplified by studies within the Hamann Group: (1) the identification of a new species of *Latrunculia* from Alaska amongst several other congeners and other species, and the isolation of two new, and six known pyrroloiminoquinones that exhibited anti-HCV, antimalarial and selective antimicrobial activities [21,32]; (2) the identification of *L.* (*L.*) *austini* Samaai, Kelly & Gibbons, 2006, from the Aleutian Islands, and differentiation from congeners in the region, facilitated the discovery of a new class of pyrroloiminoquinone alkaloids (aleutianamines) that exhibited selective bioactivity against pancreatic cancer cell lines. The molecule was identified and structurally elucidated with the guidance of mass spectrometry, nuclear magnetic resonance, and molecular ion networking (MoIN) analysis of data collected from the injection of both various discorhabdin standards and new extracts of Pacific *Latrunculia* species identified and dereplicated within this study [90].

Two examples of non-latrunculid genera further illustrate the importance of the general taxonomic process: (1) the dereplication of numerous deep-water sponge specimens and the discovery of several new species of *Monanchora* Carter, 1883 (order Poecilosclerida, family Crambeidae Lévi, 1963) [148] from the Aleutian Islands, separating these from the northeastern Pacific species, *Monanchora pulchra* Lambe, 1894 [149], facilitated the discovery of three metabolites that exhibited potential antiproliferative activity against two CRT-positive colon cancer cell lines [150]; (2) the elucidation of morphological and colour variation in *Acanthostrongylophora ingens* Thiele, 1899 [151] (order Haplosclerida, family Petrosiidae Van Soest, 1980) [152] and revision of the classification of the numerous specimens that have yielded manzamines and related compounds in the literature, over many years [152] facilitated the discovery of a sponge-associated bacterium that produces the manzamine class of antimalarials in *A. ingens* Thiele, 1899 [152].

While the discovery of potential microbial biosynthetic origins for many of the compounds in latrunculid and podospongid genera weakens their chemotaxonomic diagnostic value somewhat, by adding “noise”, the biosynthetic pathways, intermediary and end-products remain strong markers of the phylogeny of these sponges. Future research to increase our understanding of sponge phylogeny, of the role microorganisms play in sponge evolution, and the intricacies of production of interesting bioactive metabolites derived from both must include a comparison with the biosynthetic pathways, and their intermediary and end-products. As exemplified in Kerr and Kelly-Borges [153], we can use cladistic analysis to explore the congruence of sponge and microbiome phylogeny and the metabolic origins and biosynthetic pathways of the target compounds as a hypothesis to test phylogenetic relationships between these sponges. 

## 6. Conclusions

This systematic review has clearly highlighted the evidence that marine sponges of the family Latrunculiidae are prolific sources of numerous secondary metabolites that are structurally unique and exhibit a broad spectrum of biological activities. A comprehensive literature survey covering the chemical and biological investigations on the secondary metabolites isolated from marine sponges belonging to the family Latrunculiidae throughout 1986–2020 presented 110 naturally occurring metabolites that are categorized into two main groups. Additionally, the chemotaxonomic remarks, a brief insight of the proposed biogenetic pathway and the biosynthetic origin of the (bis)pyrroloiminoquinones are also discussed. 

While the systematics of the Latrunculiidae and related genera has presented multiple challenges, we now consider the systematics of this group to be one of the better known in the Porifera. Numerous studies have resolved issues with the use of chemotaxonomy and comparisons of living and fossil material from the South Pacific. Latrunculid sponges have very similar morphologies, but the key to identification is full characterization of the anisodiscorhabd spicule (ornamentation and dimensions). The discovery of new latrunculid sponges is still ongoing, particularly in New Zealand and South Africa. Discorhabdins are the most important chemical family reported from latrunculid sponges. However, there is some confusion as to the nomenclature of discorhabdins due to the great chemical diversity of this type of compounds. Structure elucidation of discorhabdins is challenging, given the fact that compounds from this chemical family commonly contain several heteroatoms in highly unsaturated polycyclic ring systems, hence, there have been some revisions on the 3D structures of discorhabdins. ECD spectroscopy coupled with computational methods has become an efficient strategy to determine the absolute configurations of discorhabdins. The broad spectrum in vitro bioactivities of discorhabdins, especially their anticancer activity, is another driving force for studying the chemistry of latrunculid sponges. However, many studies, particularly the earlier research, did not report in vitro potency (IC_50_ or MIC values) of latrunculid sponge metabolites towards cancer cell lines. Similarly, many studies mostly omitted general toxicity assessments against non-cancerous cell lines, hence it is difficult to judge on the selectivity of some bioactive pyrroloiminoquinone alkaloids. With the growing number of new and potent natural products from the marine environment, pre-clinical and pharmacokinetic studies on sponge-derived molecules are increasing [154]. To the best of our knowledge, however, no pharmacokinetic investigation has been carried out on any latrunculid sponge metabolite. 

The natural origin of (bis)pyrroloiminoquinone-type alkaloids is still in debate. A proposed microbial origin has promoted a series of culture-independent studies on the microbial community of latrunculid sponges, and the predominated Spirochaetae bacteria have been proposed to be responsible for the production of (bis)pyrroloiminoquinone-type alkaloids. However, culture-dependent studies of these Spirochaetae bacteria are still needed to verify the assumption, which could possibly solve the supply issue of (bis)pyrroloiminoquinones. Although facing significant headwinds for further drug development in different therapeutic areas, e.g., the supply issue, the predominated cytotoxicity, and the highly fused structure-property that challenges their total or semisynthesis, latrunculid metabolites, especially discorhabdin-related alkaloids, remain to be interesting targets for drug developments and new discoveries from marine sponges of family Latrunculiidae Topsent, 1922 to continue apace.

## Figures and Tables

**Table 1:** Discorhabdin enantiomers obtained from geographically distinct *Latrunculia* sponges collected from New Zealand (summarized from references [74,75,87]).

Collection Site	Sponge ID	Purified Compounds
Wellington	*L.* (*B.*) *wellingtonensis*	(+)-Discorhabdin A (**2**); (+)-discorhabdin-B (**3**); (−)_546_-discorhabdin D (**4**); (+)-discorhabdin G*/I (**25**); (−)-discorhabdin-H (**9**); (−)_546_-discorhabdin L (**13**); (−)-discorhabdin N (**15**); (+)-discorhabdin Q (**28**); (+)-1-thiomethyldiscorhabdin G*/I (**24**)
	*L.* (*B.*) *wellingtonensis*	(+)-Discorhabdin B (**3**); (+)-discorhabdin G*/I (**25**); (−)_546_-discorhabdin L (**13**); (+)-discorhabdin Q (**28**); (−)-discorhabdin W (**37**); (−)-16a,17a-dehydrodiscorhabdin W (**39**); (+)-3-dihydrodiscorhabdin A (**27**)
Kaikoura Coast	*L.* (*L.*) *triverticillata*	(+)-2-Hydroxydiscorhabdin D (**7**); (−)_546_-discorhabdin-D (**4**); 3-dihydrodiscorhabdin C (**26**); discorhabdin C (**1**)
	*L.* (*B.*) *kaikoura*	(+)-Discorhabdin-K (**12**)
Doubtful Sound	*L.* (*L.*) *fiordensis*	(−)-Discorhabdin B (**19**); discorhabdin C (**1**); (−)-discorhabdin-G*/I (**10**); (+)_546_-discorhabdin-L (**21**); (−)-discorhabdin-K_2_ (**22**); (+)-discorhabdin W (**36**); (+)-16a,17a-dehydrodiscorhabdin W (**38**)
Milford Sound	*L.* (*L.*) *fiordensis*	(−)-Discorhabdin-B (**19**); (−)-discorhabdin-G*/I (**10**); (+)-discorhabdin-H_2_ (**23**); (+)_546_-discorhabdin-L (**21**); (+)-discorhabdin-W (**36**)

**Table 2:** Cytotoxicity of discorhabdins P (**68**), S–U (**69**–**71**). IC_50_ values in μM.

Cell Line	68	69	70	71
P-388	0.052	3.08	>5	0.17
A-549	0.86	>5	>5	0.17
PANC-1	Not tested	2.6	0.7	0.069

## Data Availability

Not applicable.
